# Deregulation of Rab and Rab Effector Genes in Bladder Cancer

**DOI:** 10.1371/journal.pone.0039469

**Published:** 2012-06-19

**Authors:** Joel R. Ho, Elodie Chapeaublanc, Lisa Kirkwood, Remy Nicolle, Simone Benhamou, Thierry Lebret, Yves Allory, Jennifer Southgate, François Radvanyi, Bruno Goud

**Affiliations:** 1 Institut Curie, Centre de Recherche, Paris, France; 2 CNRS, UMR 144, Paris, France; 3 Jack Birch Unit of Molecular Carcinogenesis, Department of Biology, University of York, York, United Kingdom; 4 Université d'Evry, iSSB, Evry, France; 5 CNRS, UMR 8200, Institut de Cancérologie Gustave Roussy, Villejuif, France; 6 INSERM, U946, Paris, France; 7 Département d'Urologie, Hôpital Foch, Suresnes, France; 8 AP-HP, Groupe Hospitalier Henri Mondor, Plateforme de Ressources Biologiques, Département de Pathologie, Créteil, France; 9 INSERM, Unité 955, Créteil, France; Institute of Molecular and Cell Biology, Singapore

## Abstract

Growing evidence indicates that Rab GTPases, key regulators of intracellular transport in eukaryotic cells, play an important role in cancer. We analysed the deregulation at the transcriptional level of the genes encoding Rab proteins and Rab-interacting proteins in bladder cancer pathogenesis, distinguishing between the two main progression pathways so far identified in bladder cancer: the Ta pathway characterized by a high frequency of *FGFR3* mutation and the carcinoma *in situ* pathway where no or infrequent *FGFR3* mutations have been identified. A systematic literature search identified 61 genes encoding Rab proteins and 223 genes encoding Rab-interacting proteins. Transcriptomic data were obtained for normal urothelium samples and for two independent bladder cancer data sets corresponding to 152 and 75 tumors. Gene deregulation was analysed with the SAM (significant analysis of microarray) test or the binomial test. Overall, 30 genes were down-regulated, and 13 were up-regulated in the tumor samples. Five of these deregulated genes (*LEPRE1*, *MICAL2*, *RAB23*, *STXBP1*, *SYTL1*) were specifically deregulated in *FGFR3*-non-mutated muscle-invasive tumors. No gene encoding a Rab or Rab-interacting protein was found to be specifically deregulated in *FGFR3*-mutated tumors. Cluster analysis showed that the *RAB27* gene cluster (comprising the genes encoding RAB27 and its interacting partners) was deregulated and that this deregulation was associated with both pathways of bladder cancer pathogenesis. Finally, we found that the expression of *KIF20A* and *ZWINT* was associated with that of proliferation markers and that the expression of *MLPH*, *MYO5B*, *RAB11A*, *RAB11FIP1*, *RAB20* and *SYTL2* was associated with that of urothelial cell differentiation markers. This systematic analysis of Rab and Rab effector gene deregulation in bladder cancer, taking relevant tumor subgroups into account, provides insight into the possible roles of Rab proteins and their effectors in bladder cancer pathogenesis. This approach is applicable to other group of genes and types of cancer.

## Introduction

Intracellular trafficking is an essential process in eukaryotic cells. It relies on vesicular or tubular transport carriers that shuttle between cell compartments facilitating the constant exchange of proteins and lipids. Many studies have highlighted its complexity and led to the identification of a large number of proteins involved in the different steps of intracellular transport, i.e. the formation of transport carriers from donor membranes, their movement along cytoskeletal tracks and their tethering/fusion with target membranes. Small GTPases of the Rab family have emerged as key regulators of these different steps. As with other GTPases, Rab proteins cycle between an inactive GDP (guanosine diphosphate)-bound form and an active GTP (guanosine triphosphate)-bound form. The active GTP-bound form of the Rab is membrane-bound whereas hydrolysis of the GTP to GDP results in its dissociation into the cytosol. These two cycles are controlled by a complex regulatory network of proteins that includes guanine nucleotide exchange factors (GEFs), GTPase activating proteins (GAPs) and guanine nucleotide dissociation inhibitors (GDI). In their active form Rab GTPases interact with a diverse range of effector proteins, such as molecular motors, lipid kinases, tethering factors and scaffolding proteins (see [1] for review).

Recent studies have found a role for a number of Rab proteins in human cancers. Several expression studies have suggested that they could play both an activating and an inhibiting role in tumor progression. *RAB1A* is overexpressed in tongue squamous cell carcinoma [2]. *RAB3A* is expressed in insulinoma, but not in normal pancreatic islet cells [3]. *RAB11A* and *RAB20* expression is increased during skin carcinogenesis [4] and in exocrine pancreatic adenocarcinomas [5], respectively. By contrast, *RAB37* is down-regulated in metastatic tumors of lung cancer [6]. Both *RAB5A* and *RAB7* were shown to be up-regulated in autonomous thyroid adenomas, such an up-regulation being correlated with an accelerated thyroglobulin endocytosis and hormone production [7].

Several functional studies have confirmed the role of Rab proteins in cancer progression. RAB5A, overexpressed in hepatocellular carcinomas, seems to be determinant for liver cancer progression, as suggested by the finding that a dominant negative form of RAB5A attenuates EGF-mediated signalling and cell migration of a human hepatoma cell line [8]. Other results have shown that *RAB23*, amplified and overexpressed in diffuse-type gastric cancer, acts as an invasion mediator gene [9]. RAB25 plays a role in the development of both ovarian and breast cancers [10], [11]. However, an opposite role of *RAB25* has also been documented, i.e. as a tumor suppressor gene for colon cancer [12]. In addition, some proteins involved in Rab cycle regulation have also been implicated in carcinogenesis. For example *RIN1*, coding for a RAB5 GEF, was shown to be a breast tumor suppressor gene [13], whereas *TBC1D3B*, coding for a RAB5 GAP, was shown to be an oncogene amplified in prostate cancer [14].

Urinary bladder cancer is the fourth most common cancer in both European and American men. In women, it is the ninth most common cancer in the USA and the 14^th^ most common cancer in Europe [15], [16]. According to the stage, at first presentation, about 50% of bladder carcinoma are Ta tumors which are generally of low grade (Ta tumors are papillary tumors that do not invade beyond the basement membrane), 20% are T1 tumors (tumors which invade beyond the basement membrane but not the underlying muscularis propria) and 30% are muscle-invasive tumors (T2–4). Carcinoma *in situ* (Cis) consisting of flat, high-grade lesions not invading beyond the basement membrane are rarely found in isolation. Instead, Cis is predominantly encountered with other urothelial tumors. Clinical and molecular evidence suggest that bladder tumors arise and progress along two main pathways: the “Ta” pathway and the “carcinoma *in situ*” pathway. Ta tumors display a high recurrence rate (60%, [17]), but have a low probability (5–10%) of progressing to T1 tumors and then to muscle-invasive tumors (T2–4). By contrast, Cis often progress (in about 50% of cases), to T1 tumors and then to muscle-invasive tumors ([18] for review). The Ta pathway is characterized by a high frequency of activating mutations of the *FGFR3* gene (encoding the tyrosine kinase fibroblast growth factor receptor 3). *FGFR3* mutations are present in 70–75% of Ta tumors and absent from Cis. Their relatively low frequency in T1 (20%) and T2–4 tumors (10–15%) is consistent with the high rate of Cis progression and the low rate of Ta progression [19]–[24].

The goal of this work was to perform a systematic study to identify Rab proteins and Rab-interacting proteins whose expression is deregulated during the Ta and Cis pathways of bladder tumor pathogenesis at the transcriptomic level.

## Results

**Figure 1:**
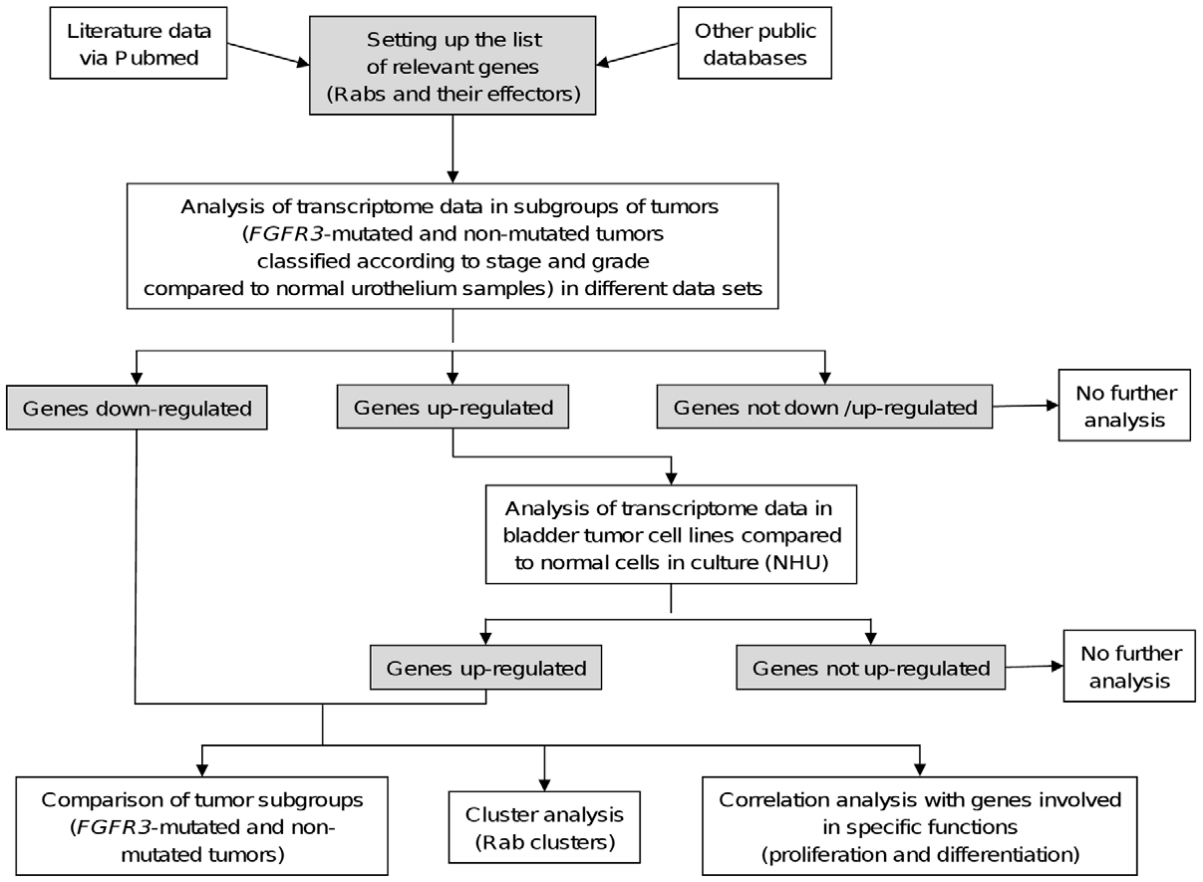
Flow chart of the different analysis steps. The first step is the identification through public data bases and expert knowledge of the genes of interest to study, here the Rabs and their effectors. The second step consists of selecting subgroups of tumors and analysing the expression of the different genes selected in the first step in these subgroups compared to the normal urothelium. The subgrouping here has been done taking into account the *FGFR3* mutation status, the stage and the grade, separating the tumors into two pathways. A comparison of the expression observed in bladder cancer cell lines and in cultured normal human urothelial cells allowed discarding of genes for which the expression could be possibly due to the presence of stroma (in comparison to normal cells, upregulation in bladder tumors but not in bladder tumor cell lines). Different types of analysis were then performed on the selected deregulated genes: 1) a comparison of the expression in *FGFR3*-mutated tumors and *FGFR3*-non-mutated tumors allowed the identification of genes specifically deregulated in one of the two pathways of bladder cancer pathogenesis; 2) by grouping genes into cluster of genes (here the Rab clusters), we identified clusters with deregulated expression; 3) by analysing the possible correlation between the expression of the deregulated genes and the expression of proliferation or differentiation marker genes, we identified the deregulated genes associated with proliferation or differentiation.

For this study, we applied the strategy presented in Figure 1 (flow chart). Briefly: 1. an exhaustive list of genes coding for Rab and Rab-interacting was established from public databases, 2. deregulated genes (up- or down-regulated compared to normal urothelium) in each of the two pathways (the *FGFR3*-mutated tumor pathway and the *FGFR3*-non-mutated tumor pathway) were identified from two transcriptome datasets, 3. deregulated genes possibly because of tumor stroma or tumor – stroma interactions were identified and then omitted from further analysis (analysis of transcriptome data in bladder tumor cell lines compared to normal cells in culture (NHU)), 4. for each deregulated gene, a specific association either with the *FGFR3*-mutated tumor group or the non-mutated tumor group was investigated as well as an association with a differentiation or proliferation phenotype. Additionally, we investigated for each Rab cluster (consisting of a given Rab protein and its interacting partners) whether it could be associated with bladder cancer pathogenesis.

**Figure 2:**
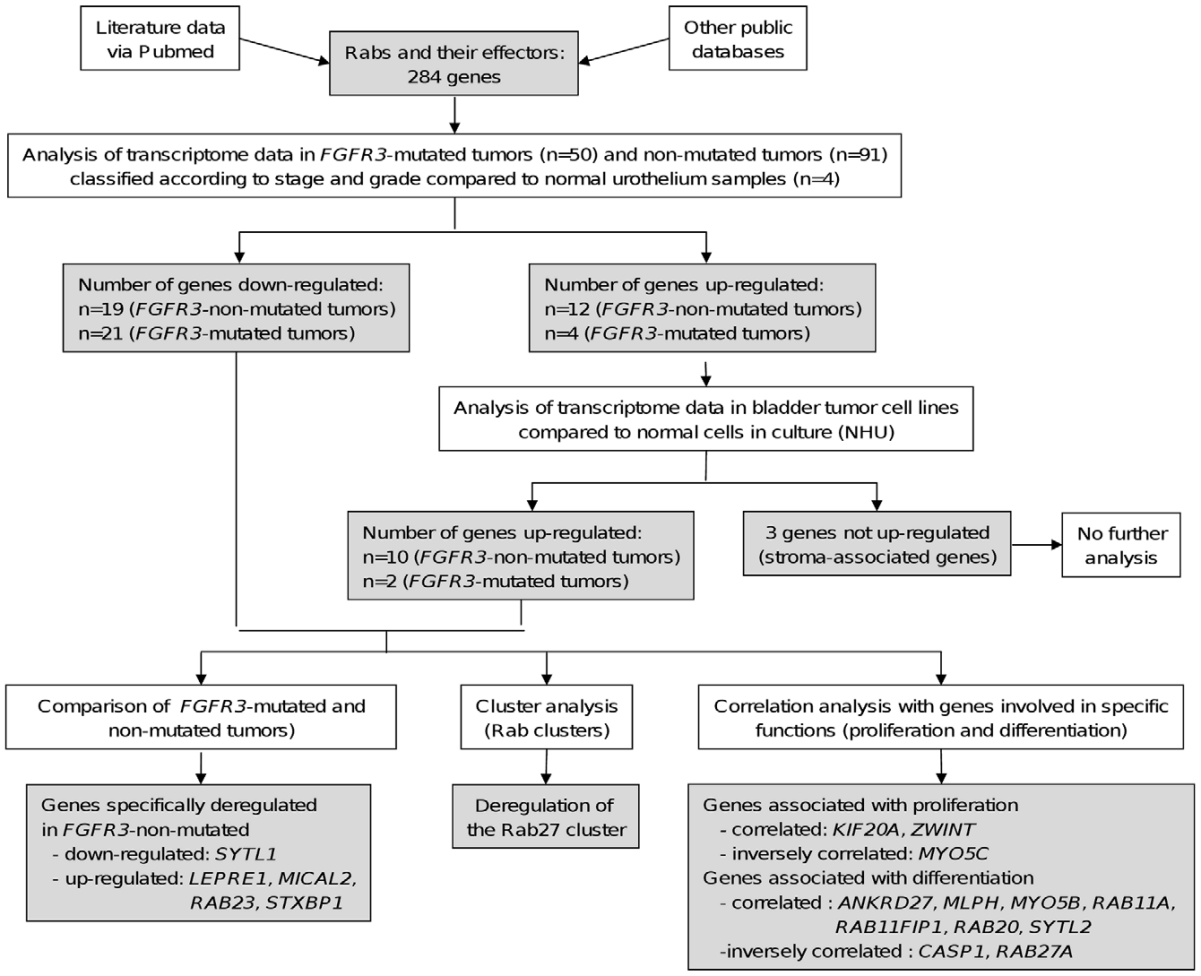
Results obtained after the different analysis steps. The results of each analysis step are shown in the flow chart presented in Figure 1.

The results obtained at each step of the analyses are summarized in Figure 2.

### Definition of the list of genes to be analysed

**Figure 3:**
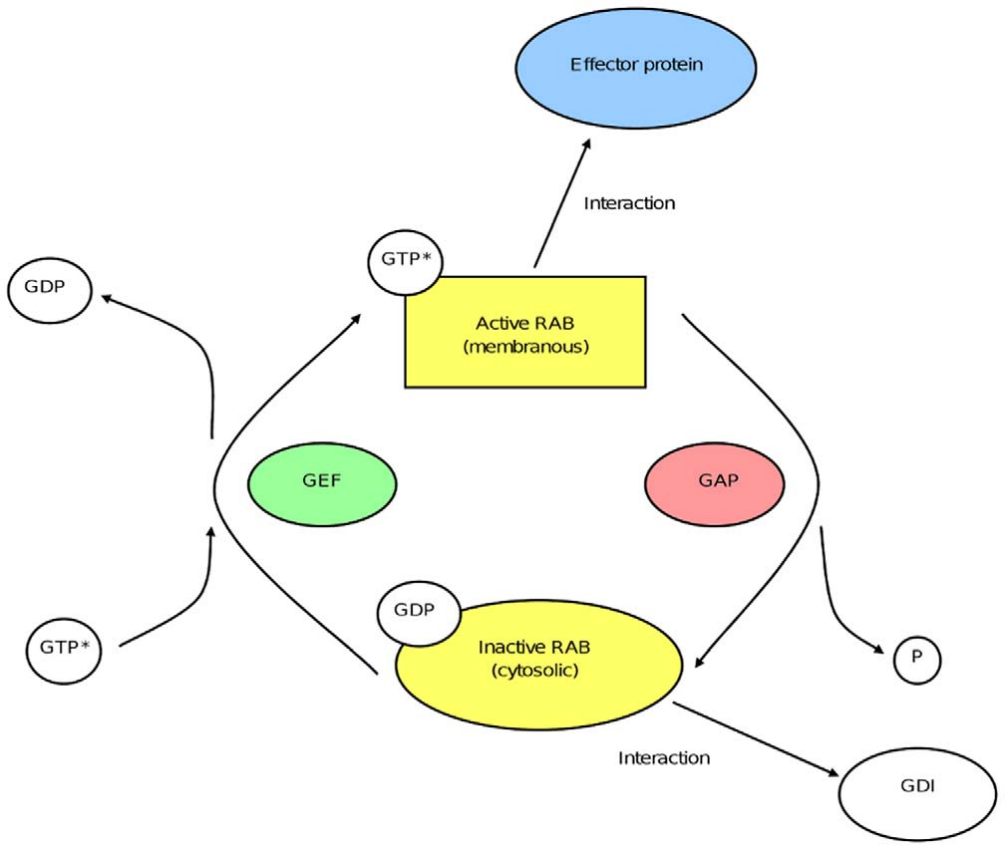
The Rab cycle. Rab GTPases cycle between an active GTP-bound form and an inactive GDP-bound form. Rab activation is mediated by a guanine exchange factor (GEF). The hydrolysis of bound GTP is catalyzed by a GTPase activating protein (GAP) resulting in the inactivation of the Rab protein. In its active form, the Rab is associated with membranes and can interact with a variety of effector proteins. In its inactive form the protein is cytosolic and is in complex with a GDP dissociation inhibitor (GDI) protein.

The first part of this work consisted of establishing a list of genes coding for Rab proteins and Rab-interacting proteins, including activating GEF and inactivating GAP proteins (Figure 3). 61 human *RAB* genes were found. The list of Rab-interacting proteins was established by a literature search to gather papers that have reported the identification and/or the characterization of proteins that directly interact with Rab GTPases, using different approaches (two-hybrid assays, GST-pull down, coimmunoprecipitation, etc.). This list comprised 217 proteins: 23 GEFs, 20 GAPs and 174 effector proteins. We added to this list two genes coding for the two GDI (GDP Dissociation Inhibitor) proteins (*GDI1* and *2*) that are common to all Rab GTPases. We also included four genes coding for proteins involved in post-translational modification and membrane association of all newly-synthesized Rabs: *CHM* and *CHML* coding for Rab escort proteins (REP) and the two isoforms of the Rab geranylgeranyl transferase (*RABGGT A* and *B*). This made a total of 284 Rabs and Rab-interacting proteins (Figure 2).

**Figure 4:**
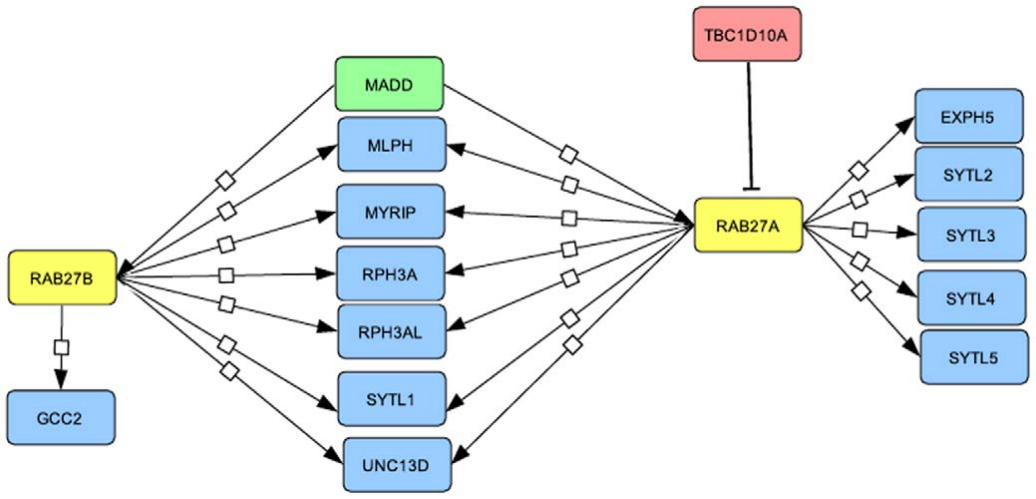
Rab and Rab-interacting proteins. Example of the Rab27 cluster. The Rab27 cluster is comprised of the two *RAB27* isoforms (*RAB27A* and *RAB27B*), the GEF *MADD*, the GAP *TBC1D10A* and 12 effector proteins. The other Rab and Rab-interacting proteins are shown in supplementary Figure S1.

The list of genes analysed in this study and the list of papers in which they were originally described are shown in Table 1 and Table S1. Figure 4 illustrates the cluster of proteins shown to interact with RAB27A/B (as an example) and Figure S1 illustrates all Rab clusters analysed; Rab proteins are in yellow, GEFs in green, GAPs in light red and effector proteins in blue.

**Table 1:** Number of isoforms, GEFs, GAPs and effector proteins analysed for each Rab protein.

	Isoform	GEF	GAP	Effector
RAB1	2	8	1	18
RAB2	2	0	3	6
RAB3	4	3	3	23
RAB4	2	0	2	19
RAB5	3	7	5	28
RAB6	3	0	2	24
RAB7	2	1	1	14
RAB8	2	2	2	22
RAB9	2	0	0	9
RAB10	1	1	3	9
RAB11	2	0	3	29
RAB12	1	0	0	1
RAB13	1	0	0	10
RAB14	1	0	1	5
RAB15	1	0	0	6
RAB17	1	0	1	1
RAB18	1	0	0	0
RAB19	1	0	0	3
RAB20	1	0	0	1
RAB21	1	2	1	0
RAB22	1	1	2	5
RAB23	1	0	1	0
RAB24	1	0	0	2
RAB25	1	0	0	7
RAB26	1	0	0	1
RAB27	2	1	1	12
RAB28	1	0	0	0
RAB30	1	0	0	3
RAB31*	1	1	0	3
RAB32	1	0	0	2
RAB33	2	0	0	8
RAB34	1	0	1	2
RAB35	1	3	1	5
RAB36	1	0	1	6
RAB37	1	0	0	2
RAB38	1	0	0	1
RAB39	2	0	1	3
RAB40	3	0	0	2
RAB41	1	0	1	3
RAB42	1	0	0	0
RAB43	1	0	1	0
RAB44	1	0	0	0

* RAB31 = RAB22B.

Some GEFs, GAPs and effector proteins are common to several Rab proteins (see Table S1 for details). *GDI1*, *GDI2*, *CHM*, *CHML*, *RABGGTA/B*, common to all Rab proteins were assessed but are not included in the count presented in this Table.

### Model of bladder cancer pathogenesis used in this study

**Figure 5:**
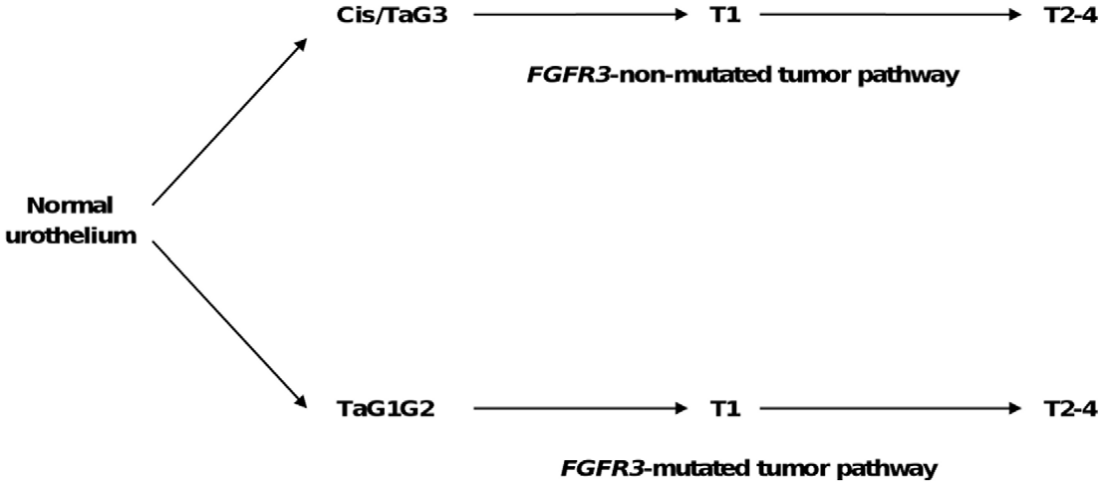
Model of bladder cancer. The “FGFR3 model” of bladder cancer progression distinguishes a *FGFR3*-non-mutated tumor pathway and a *FGFR3-*mutated tumor pathway. Cis: Carcinoma *in situ*. Stages were defined by the 1997 TNM classification and grades by the 1973 World Health Organization classification (see Material and Methods for reference).

Two main progression pathways have been so far identified in bladder cancer, the Ta pathway characterized by a high frequency of *FGFR3* mutation and the carcinoma *in situ* (Cis) pathway where no or infrequent *FGFR3* mutations have been identified. In this study we therefore considered two pathways: the *FGFR3*-mutated tumor pathway and the *FGFR3*-non-mutated tumor pathway (Figure 5). The *FGFR3*-mutated tumor pathway comprised the TaG1 and TaG2 *FGFR3*-mutated tumors, the T1 *FGFR3*-mutated tumors and the muscle-invasive *FGFR3*-mutated tumors (T2–4 tumors). We analysed two sets of bladder tumors (n = 152 in the first data set and n = 75 in the second data set). The number of *FGFR3*-mutated TaG3 was too small to identify them as a separate group (2 tumors in the first data set and 1 tumor in the second data set), but they could not either be included in the TaG1/TaG2 group due to their different clinical and molecular characteristics so they were not considered in the analysis. The *FGFR3*-non-mutated tumor pathway comprised the TaG3 *FGFR3*-non-mutated tumors, the T1 *FGFR3*-non-mutated tumors and the muscle-invasive *FGFR3*-non-mutated tumors. Transcriptomic data from Cis tumors were not available in our series. We used *FGFR3*-non-mutated TaG3 tumors instead of Cis. These tumors share several properties with Cis as they progress to an invasive stage [25] with a high probability (45%, [26]). In addition, both lesions are microscopically very similar at individual cell examination. TaG1/TaG2 tumors not mutated for *FGFR3* (9 samples in the first set of data, 2 samples in the second set of data) were not considered in the analysis as they do not fit in the Cis pathway: indeed these tumors are of low grade and they rarely progress to T1 and then to muscle-invasive tumors [26].

### Identification of up- or down- regulated genes

In order to identify genes up- or down-regulated during bladder cancer progression, the two pathways of the “FGFR3 model” were analyzed separately. We used the three groups, TaG3, T1, and T2–4, previously defined for the *FGFR3*-non-mutated pathway and the three groups, TaG1G2, T1, and T2–4 for the *FGFR-*mutated tumor pathway (Figure 5). For each tumor group, the expression of the Rab and Rab-interacting protein genes listed in Table S1 was compared to their expression in the normal urothelium (obtained without stroma) group using the statistical SAM test. The results were filtered with the following thresholds: Fold Change (FC) >1.5 for up-regulation (and <0.667 ( = 1/1.5) for down-regulation) and qValue <5%. We first analysed a collection of samples containing 4 normal urothelium and 141 tumor samples (see Material and Methods) using the Affymetrix HG U133 Plus 2.0 DNA microarrays. 269 of the 284 genes listed in Table S1 were present in these microarrays. For the *FGFR3-*non-mutated tumor pathway, 19 genes were found to be down-regulated and 12 genes up-regulated (Table 2); for the *FGFR-*mutated tumor pathway, 21 genes were found to be down-regulated and 4 genes up-regulated (Table 3) (summary in Figure 2).

**Table 2:** Deregulated genes during bladder cancer pathogenesis in the *FGFR3*-non-mutated tumor pathway.

		TaG3/normal		T1/normal		T2-4/normal	
		FC	q-value (%)	FC	q-value (%)	FC	q-value (%)
**1**	***CASP1***	0.242	18.02	**0.256**	**2.06**	0.435	9.06
**2**	***CD2AP***	0.751	56.59	0.849	44.28	0.632	3.68
**3**	***EEA1***	0.495	12.50	0.688	11.55	0.604	2.68
**4**	***GCC2***	**0.621**	**3.12**	0.751	30.32	0.610	3.04
**5**	***ICA1***	0.695	46.67	**0.618**	**3.35**	0.435	1.31
**6**	***MLPH***	0.164	7.16	**0.271**	**1.15**	0.222	2.16
**7**	***MYO5B***	0.847	79.95	0.611	23.10	0.395	4.34
**8**	***MYO5C***	0.263	46.67	0.502	5.51	0.340	3.04
**9**	***PIGR***	0.195	23.76	**0.305**	**4.46**	0.246	1.76
**10**	***RAB11A***	0.859	76.84	0.678	17.40	0.498	1.41
**11**	***RAB14***	0.746	77.98	**0.631**	**1.50**	0.591	1.31
**12**	***RAB38***	0.157	6.89	**0.277**	**1.15**	0.405	21.73
**13**	***RAB4A***	0.693	19.62	0.764	27.99	**0.541**	**3.68**
**14**	***RPH3AL***	0.566	26.01	**0.575**	**4.46**	**0.486**	**1.41**
**15**	***SYTL1***	0.746	67.80	0.498	19.07	**0.339**	**2.68**
**16**	***SYTL2***	**0.296**	**3.01**	**0.239**	**0.48**	**0.190**	**0.91**
**17**	***TBC1D30***	0.410	10.31	**0.506**	**3.35**	**0.413**	**3.68**
**18**	***TBC1D4***	0.617	15.50	**0.437**	**3.35**	0.559	10.76
**19**	***TRAPPC1***	0.545	12.50	**0.527**	**4.46**	0.634	12.73
**20**	***CAV1***	4.566	40.49	**4.912**	**0**	**10.843**	**0**
**21**	***ITGA5***	1.808	26.01	**1.874**	**0.60**	**2.848**	**0**
**22**	***KIF20A***	4.050	53.97	**4.024**	**4.46**	3.700	5.43
**23**	***LEPRE1***	1.230	62.12	**1.629**	**0.77**	**2.183**	**0**
**24**	***MICAL1***	1.244	59.20	1.237	50.76	**1.783**	**4.34**
**25**	***MICAL2***	1.224	64.43	1.478	7.55	**2.139**	**2.16**
**26**	***RAB23***	0.892	79.95	1.483	33.03	**2.674**	**2.68**
**27**	***RAB31***	1.027	76.84	1.124	62.36	**2.801**	**2.68**
**28**	***RABAC1***	1.096	76.84	1.197	53.64	**1.861**	**4.34**
**29**	***STXBP1***	0.866	79.95	1.608	35.78	**3.444**	**2.16**
**30**	***TMEM22***	1.810	31.25	1.646	21.05	**2.399**	**3.04**
**31**	***ZWINT***	3.206	49.74	**3.494**	**3.35**	**3.379**	**3.68**

From line 1 to line 19, genes are down-regulated. From line 20 to line 31, genes are up-regulated. The results in bold passed the thresholds: FC<0.667 (for down-regulation) or >1.5 (for up-regulation) and q-value <5%.

FC: Fold Change.

Number of FGFR3-non-mutated tumor samples: 3 TaG3, 25 T1, 63 T2–4.

Number of normal urothelial samples: 4.

**Table 3:** Deregulated genes during bladder cancer pathogenesis in the *FGFR3*-mutated tumor pathway.

		TaG1G2/normal		T1/normal		T2–4/normal	
		FC	q-value (%)	FC	q-value (%)	FC	q-value (%)
**1**	***ANKRD27***	**0.648**	**1.64**	0.769	32.48	0.790	38.11
**2**	***EEA1***	**0.574**	**0**	0.691	4.55	0.748	23.80
**3**	***GNAL***	0.699	0.30	**0.661**	**2.35**	0.745	23.80
**4**	***ICA1***	0.833	26.37	**0.666**	**2.88**	0.570	10.45
**5**	***MLPH***	**0.332**	**0.77**	**0.302**	**2.35**	**0.230**	**0.76**
**6**	***MYO5B***	**0.611**	**4.35**	0.552	8.65	0.492	12.88
**7**	***MYO5C***	**0.312**	**0.36**	**0.289**	**1.13**	0.374	12.88
**8**	***PIGR***	**0.216**	**0**	**0.265**	**4.55**	0.274	5.46
**9**	***RAB11FIP1***	**0.539**	**0.60**	0.561	10.72	0.505	8.38
**10**	***RAB11FIP2***	**0.572**	**3.25**	0.578	8.65	**0.521**	**1.63**
**11**	***RAB14***	**0.587**	**0.30**	**0.536**	**2.35**	**0.593**	**1.69**
**12**	***RAB20***	**0.513**	**0.60**	**0.484**	**4.55**	0.435	5.46
**13**	***RAB27A***	**0.542**	**0.30**	0.664	40.85	1.126	70.25
**14**	***RAB27B***	**0.486**	**0**	0.653	17.18	0.469	1.16
**15**	***RAB8B***	**0.583**	**0.77**	0.766	62.91	**0.647**	**23.80**
**16**	***RAB9A***	**0.617**	**0.36**	**0.582**	**2.88**	0.591	4.45
**17**	***RABGAP1L***	**0.619**	**4.35**	0.641	40.85	1.156	70.25
**18**	***RPH3AL***	0.696	1.64	0.540	7.07	**0.496**	**1.63**
**19**	***SYTL2***	**0.303**	**0.19**	**0.273**	**0.31**	**0.264**	**1.69**
**20**	***TBC1D30***	**0.459**	**0.19**	**0.492**	**1.34**	**0.427**	**1.63**
**21**	***UNC13B***	**0.572**	**0.77**	0.615	7.07	**0.502**	**1.63**
**22**	***CAV1***	**2.603**	**2.51**	3.681	5.82	**4.715**	**3.39**
**23**	***ITGA5***	**1.570**	**0.36**	**1.826**	**2.88**	2.068	10.45
**24**	***MICAL1***	**1.726**	**1.64**	1.478	12.27	1.487	43.39
**25**	***SDC1***	**1.621**	**3.25**	1.387	22.11	1.525	43.39

From line 1 to line 21, genes are down-regulated. From line 22 to line 25, genes are up-regulated. The results in bold passed the thresholds: FC<0.667 (for down-regulation) or >1.5 (for up-regulation) and q-value <5%.

FC: Fold Change.

Number of FGFR3-mutated tumor samples: 28 TaG1G2, 13 T1, 9 T2–4.

Number of normal urothelial samples: 4.

We then analysed a second set of tumors to verify the results obtained. This set, collected independently from the first set, was analysed with the Affymetrix HG U95A/U95Av2 DNA microarrays and contained 5 normal urothelium and 72 tumor samples (see Material and Methods). Only 165 of the 284 genes listed in Table S1 (58 %) were present in these microarrays. Among the 31 genes found to be up- or down-regulated in the *FGFR3-*non-mutated tumor pathway (Table 2), 17 were present on the Affymetrix HG U95A/U95Av2 DNA microarrays: *CASP1*, *CAV1*, *CD2AP*, *EEA1*, *ICA1*, *ITGA5*, *MICAL2*, *PIGR*, *RAB11A*, *RAB14*, *RAB31*, *RAB4A*, *RABAC1*, *STXBP1*, *TBC1D30*, *TBC1D4* and *ZWINT*. Among the 25 genes found to be up- or down-regulated in the *FGFR3-*mutated tumor pathway (Table 3), 14 were present on the Affymetrix U95A/U95Av2 DNA microarrays: *CAV1*, *EEA1*, *ICA1*, *ITGA5*, *PIGR*, *RAB11FIP2*, *RAB14*, *RAB27A*, *RAB27B*, *RAB9A*, *RABGAP1L*, *SDC1*, *TBC1D30* and *UNC13B*. For these genes, 56 % of the results obtained with the first set of data were validated with the second set of data with at least one threshold passed: FC>1.5 (or <0.667) and/or qValue <5% (Table S2). The other results were not significant. Of note, no discordant result between the two sets of data was found.

### Expression analyses of upregulated genes in vitro

A tumor is composed of both tumor and stromal cells. As the transcriptomic analysis is performed with this mix of cells, up-regulation of a given gene could then be due to the presence of stroma (genes up-regulated in the stroma or in the tumor cells due to stromal cell – tumor cell interaction).

**Figure 6:**
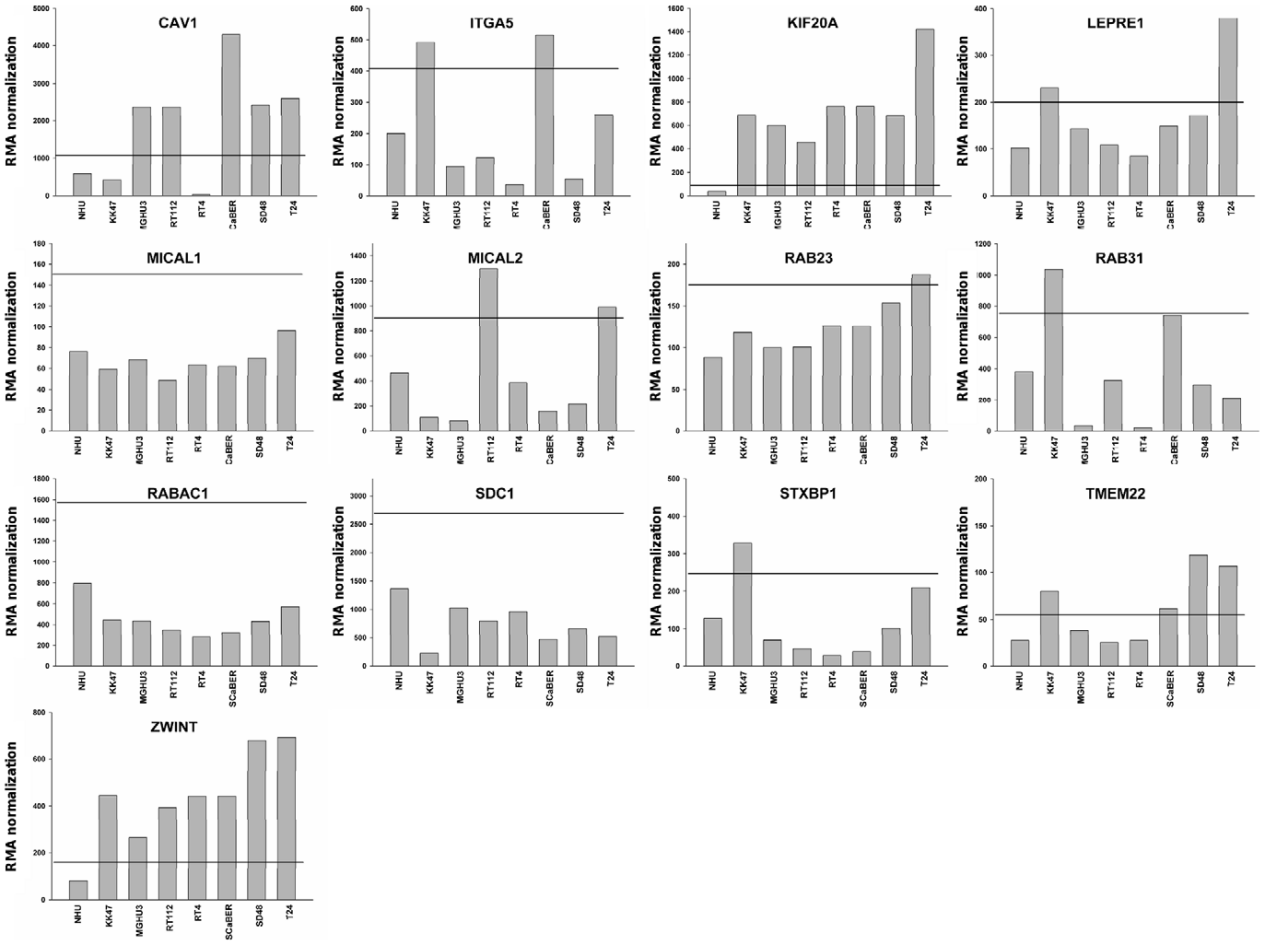
Up-regulated gene expression in normal human urothelium (NHU) cells and bladder tumor cell lines. The expression of the 13 up-regulated genes (Table 2 and 3) (*CAV1*, *ITGA5*, *KIF20A*, *LEPRE1*, *MICAL1*, *MICAL2*, *RAB23*, *RAB31*, *RABAC1*, *SDC1*, *STXBP1*, *TMEM22* and *ZWINT*) was measured by Affymetrix array in 7 bladder cancer cell lines (KK47, MGHU3, RT112, RT4, SCaBER, SD48 and T24) and normal human urothelial (NHU) cells grown in culture. The threshold for genes to be considered as up-regulated in tumor cell lines (2 fold the expression measured in NHU cells) is represented by a black line.

To address this issue, we analyzed in normal human urothelial (NHU) cells grown in culture under regenerating and differentiating conditions [27], [28] and in 7 established bladder cancer cell lines (without stromal cells) the expression of the 13 genes found to be up-regulated in the *FGFR3-*non-mutated and mutated tumor pathways: *CAV1*, *ITGA5*, *KIF20A*, *LEPRE1*, *MICAL1*, *MICAL2*, *RAB23*, *RAB31*, *RABAC1*, *SDC1*, *STXBP1*, *TMEM22* and *ZWINT* (Table 2 and Table 3). For most of the genes, we found at least one cancer cell line in which its expression was at least two-fold higher than in cultured normal urothelial cells. This was not the case for *MICAL1*, *RABAC1* and *SDC1* (Figure 6). The overexpression of *MICAL1* and *RABAC1* is likely due to the presence of stromal cells in the tumor as *MICAL1* is highly expressed in different types of hematopoietic cells (dendritic cells, mast cells and NK cells) and *RABAC1* in mast cells (data not shown). The overexpression of *SDC1* could be due to stromal-epithelial interaction. *MICAL1*, *RABAC1* and *SDC1* were excluded for further analysis (see summary in Figure 2).

### Genes specifically up- or down-regulated in each pathway

**Figure 7:**
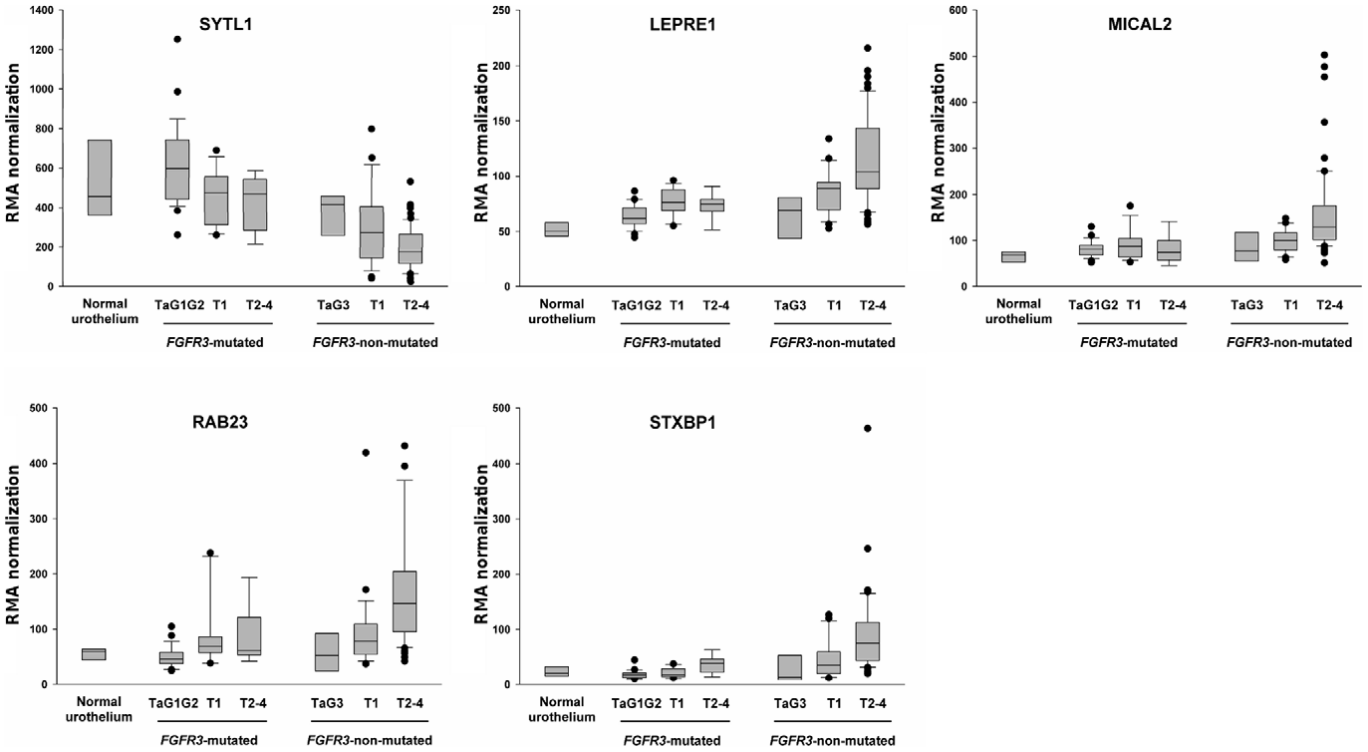
Expression in normal samples and in the two groups of tumors (mutated and non-mutated for *FGFR3*) classified according to stage, of the genes found to be specifically deregulated in one of the two pathways of bladder tumor pathogenesis. Expression of *SYTL1*, *LEPRE1*, *MICAL2*, *RAB23* and *STXBP1* measured by Affymetrix array in normal samples (n = 4) and *FGFR3*-mutated tumor samples (TaG1G2, n = 28; T1, n = 13; T2-4, n = 9), and *FGFR3-*non-mutated samples (TaG3, n = 3; T1, n = 25; and T2-4, n = 63). Are represented the 10th percentile (below bar), the 25th percentile (box bottom), the median (bar in the box), the 75^th^ percentile (box top) and the 90^th^ percentile (upper bar). The points represent the outlier samples. *SYTL1* is down-regulated in *FGFR3*-non-mutated tumors, whereas *LEPRE1*, *MICAL2*, *RAB23* and *STXBP1* are up-regulated.

Among the genes found to be deregulated during bladder cancer progression (Table 2 and Table 3), 13 were common to both pathways, 10 down-regulated: *EEA1*, *ICA1*, *MLPH*, *MYO5B*, *MYO5C*, *PIGR*, *RAB14*, *RPH3AL*, *SYTL2*, *TBC1D30* and 3 up-regulated: *CAV1*, *ITGA5*, *MICAL1*. In order to search for genes specifically deregulated in each pathway, we proceeded in two steps. 1. We selected the genes found significantly up- or down- regulated only within the *FGFR3-*non-mutated tumor pathway or within the *FGFR3*-mutated tumor pathway. 9 down-regulated and 8 up-regulated genes were selected for the *FGFR3*-non-mutated tumor pathway and 11 down-regulated genes were selected for the *FGFR3-*mutated tumor pathway (Table S3, left column). 2. We used the statistical SAM test to compare the expression of the selected genes in the tumor group of the same stage but of opposite *FGFR3* mutation status (Table S3, right column). *SYTL1* was significantly down-regulated in the T2–4 (*FGFR3*-non-mutated) tumor group whereas *LEPRE1*, *MICAL2*, *RAB23* and *STXBP1* were significantly up-regulated in the same tumor group (Table 4 and Figure 7; see summary in Figure 2). No gene was found to be specifically deregulated for the *FGFR3-*mutated tumors.

**Table 4:** Genes specifically deregulated in one of the two pathways of bladder cancer pathogenesis.

	T2-4 FGFR3-non-mutated tumors/normal		T2-4 FGFR3-non-mutated tumors/T2–4 FGFR3-mutated tumors	
	FC	q-value (%)	FC	q-value (%)
***SYTL1***	0.339	2.68	0.418	1.72
***LEPRE1***	2.183	0	1.528	1.24
***MICAL2***	2.139	2.16	1.804	1.24
***RAB23***	2.674	2.68	1.920	4.08
***STXBP1***	3.444	2.16	2.314	2.53

SAM analysis of the deregulated genes was performed comparing their expression, for a given stage, *FGFR3*-mutated tumors and *FGFR3*-non-mutated tumors. Only the genes found differentially expressed are presented. *SYTL1*, which is down-regulated in *FGFR3*-non-mutated T2–4 tumors compared to normal urothelium (first 2 columns and Table 2) is also specifically down-regulated in *FGFR3*-non-mutated T2–4 tumors compared to *FGFR3*-mutated T2–4 tumors (last 2 columns). *LEPRE1*, *MICAL2*, *RAB23* and *STXBP1*, which are up-regulated in *FGFR3*-non-mutated T2–4 tumors compared to normal urothelium (first 2 columns and Table 2) are also specifically up-regulated in *FGFR3*-non-mutated T2–4 tumors compared to *FGFR3*-mutated T2–4 tumors (last 2 columns). FC: Fold Change.

### Analysis by clusters of genes

In the previous part of this study, we used the statistical SAM test to analyse separately the expression of each gene during bladder cancer progression. To investigate whether a Rab cluster (consisting of a given Rab protein and its interacting partners, see Figure 4 and Figure S1) could be specifically associated with bladder cancer progression, we used the statistical binomial test to determine whether the percentage of genes deregulated within a given Rab cluster was significantly greater than the percentage obtained by analyzing all the Rab and Rab-interacting protein genes. This test was applied for each Rab cluster within each tumor group and the results were filtered with the threshold pValue <1%. Interestingly, the Rab27 cluster passed this threshold for the T2–4 tumor groups of both the *FGFR3-*non-mutated and mutated tumor pathways (Table 5 and Table S4; see summary in Figure 2). In addition to *RAB27A* and *RAB27B*, the genes down-regulated in this cluster comprise *GCC2*, *MLPH*, *RPH3AL*, *SYTL1* and *SYTL2*.

**Table 5:** Deregulation of the Rab27 cluster.

		*FGFR3-*non-mutated tumor pathway						*FGFR3*-mutated tumor pathway					
		TaG3/normal		T1/normal		T2–4/normal		TaG1G2/normal		T1/normal		T2–4/normal	
		FC	q-value (%)	FC	q-value (%)	FC	q-value (%)	FC	q-value (%)	FC	q-value (%)	FC	q-value (%)
***RAB27A***		0.510	34.08	0.757	50.76	1.176	60.73	**0.542**	**0.30**	0.664	40.85	1.126	70.25
***RAB27B***		0.602	23.76	0.747	35.78	0.506	5.43	**0.486**	**0**	0.653	17.18	**0.469**	**1.16**
***MADD***	GEF	0.849	12.85	0.777	7.55	0.787	6.72	0.980	49.78	0.879	45.50	0.801	20.04
***TBC1D10A***	GAP	0.710	31.25	0.827	30.32	0.735	9.06	0.839	33.92	0.807	32.48	0.732	10.45
***EXPH5***	Eff	1.597	62.12	1.039	68.42	0.826	58.06	0.587	10.22	0.702	22.11	0.594	8.38
***GCC2***	Eff	**0.621**	**3.12**	0.751	30.32	**0.610**	**3.04**	0.890	44.88	0.776	24.58	0.780	43.39
***MLPH***	Eff	0.164	7.16	**0.271**	**1.15**	**0.222**	**2.16**	**0.332**	**0.77**	**0.302**	**2.35**	**0.230**	**0.76**
***MYRIP***	Eff	1.131	67.80	1.154	25.69	1.323	42.78	1.146	14.11	1.236	40.85	1.148	15.62
***RPH3AL***	Eff	0.566	26.01	**0.575**	**4.46**	**0.486**	**1.41**	0.696	1.64	0.540	7.07	**0.496**	**1.63**
***SYTL1***	Eff	0.746	67.80	0.498	19.07	**0.339**	**2.68**	1.182	26.37	0.891	68.81	0.812	75.24
***SYTL2***	Eff	**0.296**	**3.01**	**0.239**	**0.48**	**0.190**	**0.91**	**0.303**	**0.19**	**0.273**	**0.31**	**0.264**	**1.69**
***SYTL3***	Eff	0.806	12.50	0.728	3.83	0.821	24.09	0.724	0.60	0.776	3.73	0.768	12.88
***SYTL4***	Eff	2.389	56.59	0.985	72.47	0.669	39.61	0.678	26.37	0.761	68.07	0.861	80.81
***SYTL5***	Eff	0.822	79.64	1.074	64.73	0.710	14.94	0.845	43.92	1.054	62.34	0.813	64.39
***UNC13D***	Eff	1.005	76.84	1.136	47.28	1.069	69.16	1.275	10.22	1.093	59.96	1.017	76.63
Nb of Rab27 cluster deregulated genes		2		3		5		4		2		4	
Nb of Rab and Rab effector deregulated genes		2		16		24		20		11		10	
Binomial test (p-value)		0.005442		0.05592		0.007908		0.02145		0.1235		0.001874	

The values in bold passed the thresholds: FC<0.667 and q-value <5%. *RPH3A* (not represented, coding for a Rab27 effector protein) is a gene absent in the Affymetrix HG U133 Plus 2.0 DNA microarrays. The binomial test p-values <1% are underlined.

### Genes associated with proliferation

**Figure 8:**
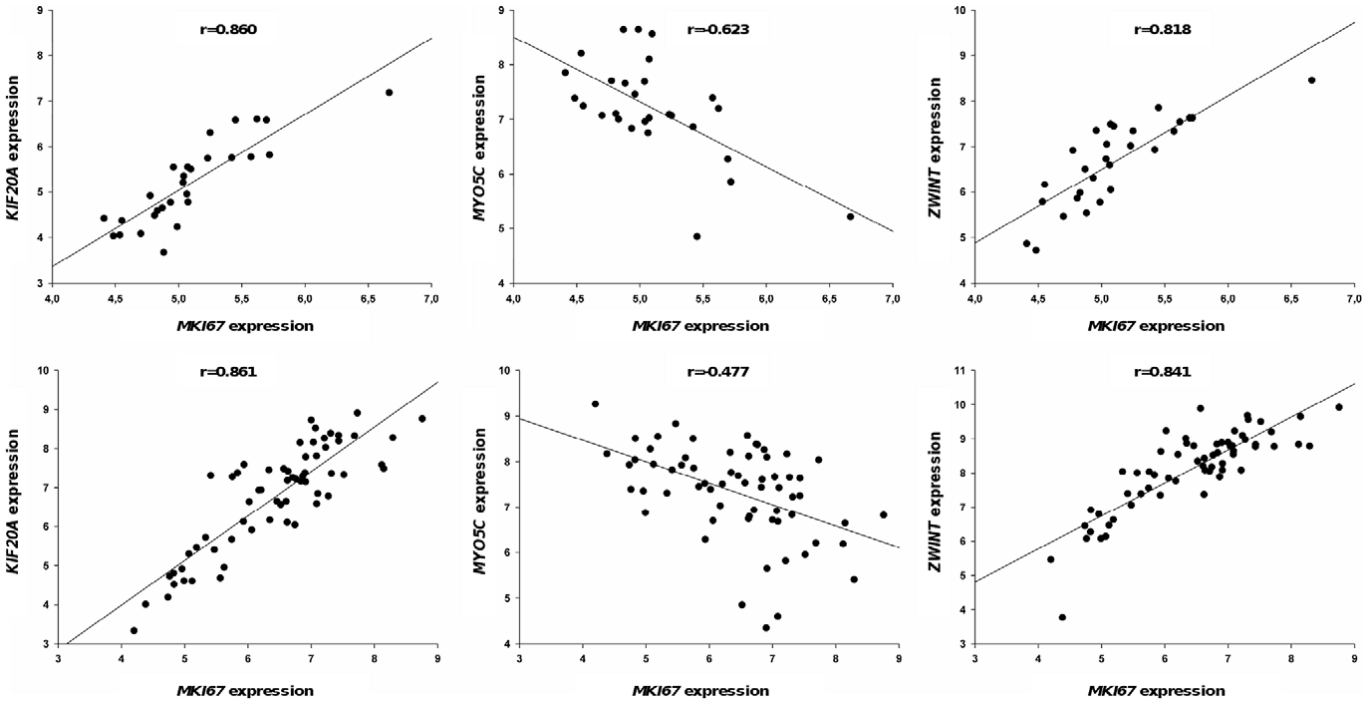
Several deregulated genes have their expression correlated with the expression of *MKI67*, a proliferation marker gene. The Pearson correlation coefficient (r) between the expression of the deregulated genes and the expression of proliferation marker gene, *MKI67*, was calculated for *FGFR3*-mutated tumors in the TaG1G2 group (n = 28) and for *FGFR3*-non-mutated tumors in the T2–4 group (n = 63). The expression of the deregulated genes as a function of *MKI67* expression is shown in TaG1G2 *FGFR3*-mutated tumors (upper figures) and in T2–4 non-mutated tumors (lower figures). Only the plots for the correlated genes are presented (p<1%, which corresponds to a correlation coefficient, |r| above 0.479 for the *FGFR3*-mutated tumor group and above 0.323 for the *FGFR3*-non-mutated tumor group).

In order to evaluate a possible association of the deregulated genes listed in Table 2 and Table 3 with cell proliferation, we calculated a Pearson correlation between their expression and that of the proliferation marker gene: *MKI67* [29]. For this analysis, among the 6 groups constituted for this study, we worked with the two homogenous tumor groups with the higher number of samples: the Ta G1/G2 (*FGFR3*-mutated) tumor group (28 samples) and the stage T2–4 (*FGFR3*-non-mutated) tumor group (63 samples). We first noticed, as expected, that the expression of *MKI67* was significantly higher in the T2–4 (*FGFR3* non-mutated) group than in the Ta G1/G2 (*FGFR3*-mutated) group (about 3 fold; Student test, p = 8.8E-10). We chose to filter the Pearson correlation values with the threshold: |r| >0.479 (pVal <1%) for the Ta G1/G2 (*FGFR3*-mutated) tumor group and |r| >0.323 (pVal <1%) for the T2–4 (*FGFR3*-non-mutated) tumor group. The expression of a gene was considered to be correlated with the expression of *MKI67* if the pValue <1% with both tumor groups. The expression of *KIF20A* and *ZWINT* were correlated, whereas the expression of *MYO5C* was inversely correlated, with the expression of *MKI67* (Figure 8 and Table S5; see summary in Figure 2).

### Genes associated with differentiation

The same analysis was performed to evaluate the association of deregulated genes with the differentiation process of urothelial cells. The uroplakin genes *UPK1A*, *UPK1B*, *UPK2*, *UPK3A* and *UPK3B*, encoding for urothelium-specific markers [30]–[33] and the two genes *GRHL3* [34] and *FOXA1* [28], encoding for transcription factors, were used as markers of urothelial differentiation. We used the same two tumor groups as above. The results of the Pearson correlation were filtered with the same thresholds as above and we considered that the expression of a gene is correlated with the expression of another gene if the pValue was less than 1% for both tumor groups. The results are shown in Table 6 and Table S6. Seven genes were found to be correlated with at least one urothelial differentiation marker: *ANKRD27*, *MLPH*, *MYO5B*, *RAB11A*, *RAB11FIP1*, *RAB20* and *SYTL2*. Two genes were found to be inversely correlated with at least one urothelial differentiation marker: *CASP1* and *RAB27A* (see summary in Figure 2).

**Table 6:** Deregulated genes correlated with differentiation markers.

	FOXA1		GRHL3		UPK1A		UPK1B		UPK2		UPK3A		UPK3B	
	TaG1G2	T2–4	TaG1G2	T2–4	TaG1G2	T2–4	TaG1G2	T2–4	TaG1G2	T2–4	TaG1G2	T2–4	TaG1G2	T2–4
***ANKRD27***	0.331	0.024	**0.511**	**0.382**	**0.62**	**0.327**	0.211	0.11	**0.691**	**0.386**	0.361	0.142	0.138	0.209
***CASP1***	0.021	−0.51	−0.175	−0.193	−0.402	−0.349	−0.27	−0.158	−0.509	−0.319	−**0.482**	−**0.353**	−0.345	−0.2
***MLPH***	0.177	0.318	−0.114	−0.062	0.398	0.297	**0.559**	**0.379**	0.255	0.241	0.039	0.5	0.562	0.22
***MYO5B***	0.458	0.35	0.436	0.502	**0.593**	**0.53**	0.272	0.363	**0.583**	**0.6**	0.314	0.428	0.415	0.39
***RAB11A***	0.199	0.43	0.344	0.52	**0.508**	**0.392**	0.229	0.273	**0.604**	**0.396**	0.304	0.324	0.412	0.274
***RAB11FIP1***	**0.629**	**0.502**	0.471	0.578	**0.698**	**0.55**	0.317	0.552	**0.481**	**0.672**	0.184	0.625	0.335	0.692
***RAB20***	−0.02	0.367	−0.025	0.488	0.347	0.56	0.079	0.474	**0.574**	**0.536**	**0.488**	**0.475**	0.345	0.399
***RAB27A***	−0.011	−0.162	−0.104	−0.374	−**0.506**	−**0.325**	−0.163	−0.181	−**0.543**	−**0.372**	−0.529	−0.248	−0.067	−0.185
***SYTL2***	**0.559**	**0.48**	0.395	0.28	0.433	0.472	0.235	0.39	0.296	0.363	−0.079	0.555	−0.02	0.448

The Pearson correlation coefficient (r) of the expression of the deregulated genes with the expression of urothelial differentiation markers in *FGFR3*-mutated superficial tumors (TaG1G2) (n = 28 samples) and *FGFR3*-non-mutated muscle-invasive tumors (T2–4) (n = 63) is presented. Correlation with p<1% (|r| above 0.479 for Ta-T1 tumors, and |r| above 0.323 for *FGFR3*-non-mutated T2–4 tumors) are written in bold.

### Gene expression in NHU cells in culture

Normal human urothelial cells (NHU cells) can grow in culture for a finite number of passages until they enter into senescence. Before senescence, differentiation can be induced by growing them in specific media. Here NHU cells, after passage, were either grown in non-differentiating conditions (control) or in differentiating conditions (in the presence of an EGFR (epidermal growth factor receptor) inhibitor and an activator of PPARγααμμ (peroxisome proliferator-activated receptor gamma)) [28]. In control conditions, cells reach confluency and become contact-inhibited, whereas in differentiating conditions they also stop growing but begin to express markers of urothelial terminal differentiation, such as the uroplakins (UPKs).

**Figure 9:**
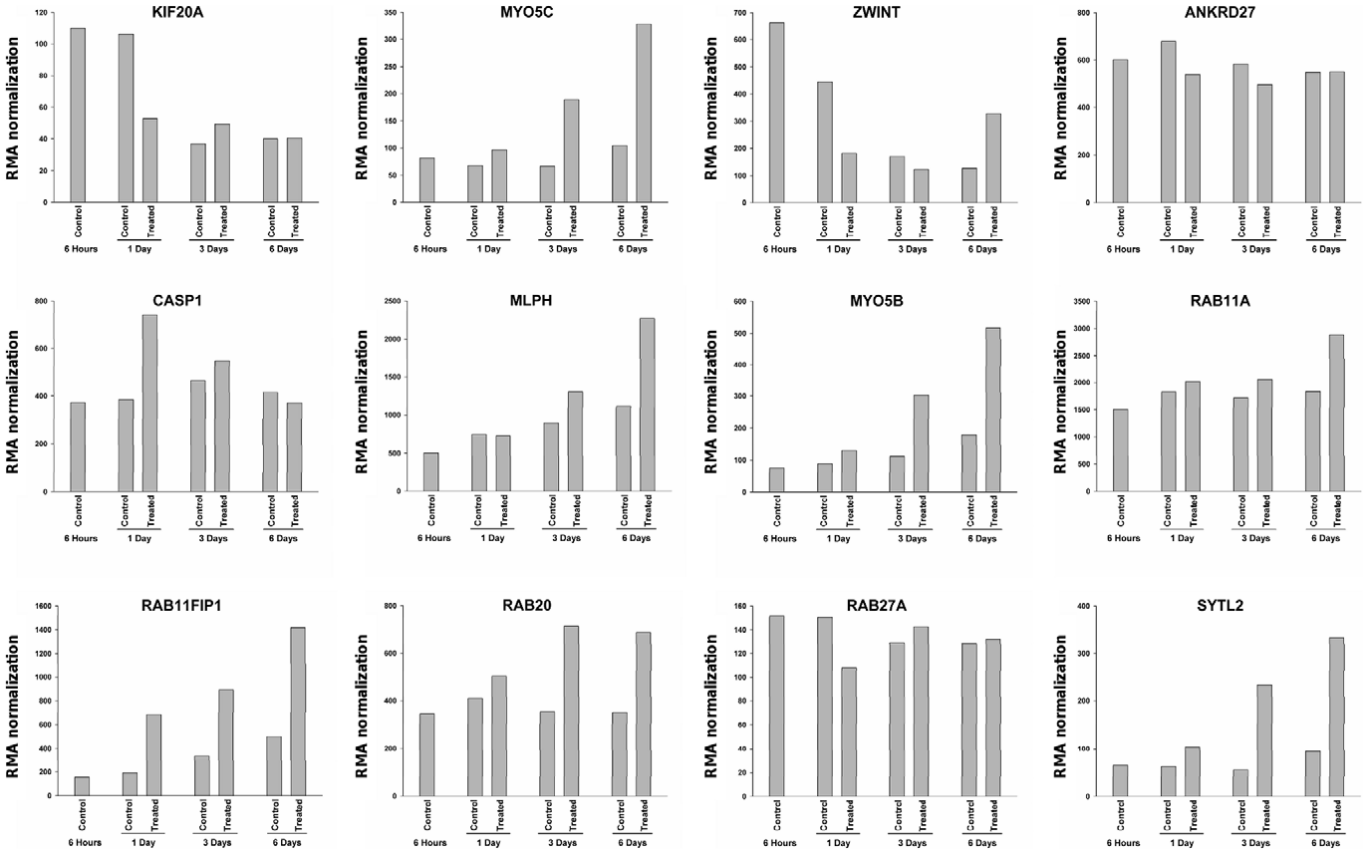
Gene expression in differentiated or non differentiated normal urothelial cells of the genes found to be associated with proliferation or differentiation in tumors. The expression of the genes found to be associated with proliferation or differentiation in tumors were measured in normal urothelial cells (NHU) in culture at four different times after passage: 6 hours, 1 day, 3 days and 6 days in differentiating conditions (in the presence of an inhibitor of EGFR and an activator of PPARγααμμ in the NHU medium) or in non-differentiating conditions (NHU medium only).

We used Affymetrix data of NHU cells in different conditions to look at the expression of the genes previously found to be associated in tumors with proliferation or differentiation markers. The NHU expression data were obtained at different times after passage: 6 hours, 1 day, 3 days and 6 days, with or without differentiating medium. As shown in Figure 9, the expression of *KIF20A* and *ZWINT* decreased in cultures treated with the differentiating medium, as well as in control cultures upon reaching confluence, indicating a link of these two genes with proliferation. In this model, *MYO5C* was not linked with proliferation, as observed in tumor expression data, but with differentiation (increased expression in the differentiating medium but not in the control medium). Among the 7 genes positively correlated with differentiation markers in the tumors, 6 were indeed induced by the differentiating medium (*MLPH*, *MYO5B*, *RAB11A*, *RAB11FIP1*, *RAB20* and *SYTL2*) but not *ANKDR27*. Neither of the two genes inversely correlated with differentiation (*CASP1* and *RAB27A*) were down-regulated upon differentiation in NHU cells.

## Discussion

Growing evidence indicates that Rab proteins and their effectors are involved in cancer progression, both as inhibitory and promoting factors. However, to our knowledge no systematic study has addressed their deregulation during cancer progression. In this study, we have identified several genes coding for Rabs and Rab-interacting proteins whose expression is deregulated during bladder cancer pathogenesis.

### Pertinence of the two pathways model

Based on clinical and molecular evidence, two progression pathways have been described in bladder cancer, the Ta pathway presenting high frequency of *FGFR3* mutation and the Cis pathway with no or low rate of *FGFR3* mutation. In this study, a simple method, i.e. the presence or absence of *FGFR3* mutation, was used to separate the tumors within the two pathways. This led to discard the TaG3 *FGFR3*-mutated tumors (4% of *FGFR3*-mutated cases in the first data set) and the TaG1, G2 *FGFR3*-non-mutated tumors (9% of *FGFR3*-non-mutated cases in the first data set), as in these two subgroups the *FGFR3* mutation was likely not the correct parameter for classifying a tumor in one of the two pathways (see paragraph on “Model of bladder cancer pathogenesis used in this study” in the Results section). Beside *FGFR3* mutation, two other molecular markers can be used to distinguish the two pathways of bladder tumor progression: the Cis signature [35] and the MRES (multiple regional epigenetic silencing) phenotype [36]. The classifications obtained by using the three different methods (Cis signature, MRES phenotype and *FGFR3* mutations), although presenting an important overlap, display some differences ([36] and data not shown). A better characterization of the *FGFR3* signaling pathway, as well as a better knowledge of the mechanism involved in the MRES phenotype and a detailed analysis of the precursor lesions will lead to a better definition of the two pathways of bladder tumor progression and possibly to the definition of additional pathways. It will be important to apply again the analyses described in this study once the different pathways will have been better characterized. Because of more homogeneous subgroups, this could lead to the identification of additional genes involved in particular subgroups of tumors.

Globally, more genes were found to be down-regulated (30) than up-regulated (13) (p = 2.16.10^−5^, binomial test). Interestingly, more genes were found to be up-regulated in the *FGFR3-*non-mutated tumor pathway (12 genes) as compared to the *FGFR3-*mutated tumor pathway (4 genes). This suggests the existence of two different mechanisms for tumor progression along the two pathways. *LEPRE1*, *MICAL2*, *RAB23* and *STXBP1* were specifically up-regulated in the muscle-invasive *FGFR3*-non-mutated tumors as compared to both normal urothelium and muscle-invasive *FGFR3*-mutated tumors, suggesting that these genes play an active role in tumor progression along the *FGFR3-*non-mutated tumor pathway. By contrast, *SYTL1* was specifically down-regulated in the muscle-invasive *FGFR3*-non mutated tumors. Thus *SYTL1* could play a role as an inhibitor of tumor progression along this pathway. Alternatively *SYTL1* could be lost because it is not essential in these tumors, but is maintained in the *FGFR3-*mutated tumors where it is essential. Overall, these results suggest that even at the muscle-invasive stage, the tumors of the two pathways should be distinguishable.

Concerning *MICAL2*, which codes for a RAB1 effector [37], this gene has been previously shown to be up-regulated in prostate cancer and to be necessary for prostate cancer cell viability [38]. *LEPRE1* encodes for an enzyme member of the collagen prolyl hydroxylase family. These enzymes are localized to the ER, and their activity is required for proper collagen synthesis and assembly. We indeed found a correlation between the expression of *LEPRE1* and the expression of *COL1A1* and *COL1A2* (data not shown). The last gene, *STXBP1*, encodes for a RAB3 effector and regulates exocytosis in neurons and neuroendocrine cells through modulation of vesicle docking and membrane fusion [39]. This suggests a possible neuroendocrine differentiation in the *FGFR3-*non-mutated tumor pathway that would not be present in the *FGFR3-*mutated tumor pathway.

### Genes deregulated in the TaG1G2 (*FGFR3*-mutated) group

Most of the deregulated genes in the *FGFR3*-mutated tumor pathway were found in this group. *ANKRD27*, *RAB20* and *RAB11FIP1* were correlated with *UPK1A, UPK2, UPK3A, FOXA1* and *GRHL3* (Table 6), suggesting that they are associated with urothelial cell differentiation. Even if the tumors of low grade (Ta)G1G2 still appeared well differentiated, we nevertheless observed a down-regulation of *UPK1A*, *UPK3A* and *GRHL3* (not *UPK2* and *FOXA1*, data not shown). *ANKRD27* encodes for a RAB21 GEF [40], a Rab involved in the early endocytic pathway [41]. ANKRD27 also interacts with VAMP7 [42], RAB32 and RAB38 [43]. Less is known about RAB20 function, a Golgi-associated Rab overexpressed in exocrine pancreatic carcinoma [5]. RAB11FIP1 (also named RCP) is a RAB11A effector [44] involved in recycling processes from endosomes to the plasma membrane. The finding that RAB11FIP1 is likely to be involved in urothelial cell differentiation is consistent with the role of RAB11A in urothelial umbrella cells [45]. Nevertheless, RAB11FIP1 also interacts with RAB25 [44] that can act as a tumor suppressor or tumor promoter depending on the RCP expression status [46]. Of note, *RAB25* was not found to be deregulated in this study. Finally, the deregulation of *RAB27A/B* suggests an alteration of the transport of lysosome-related organelles [47] in the tumors of the *FGFR-* mutated tumor pathway (see also below).

### Genes deregulated in the T2–4 (*FGFR3*-non-mutated) group

Most of the deregulated genes in the *FGFR3*-non-mutated tumor pathway were found in this group. Among them, *RAB11A* expression was correlated with the expression of *UPK1A* and *UPK2*, two urothelium marker genes also down-regulated in this tumor group (data not shown). The down-regulation of *RAB11A* may thus be secondary to the loss of differentiation. This result is consistent with the finding that the exocytosis of the discoidal/fusiform vesicles in the most differentiated urothelial cells, the umbrella cells, is RAB11A-dependent [45].

The expression of *ZWINT* was found to be correlated with the expression of *MKI67*, a proliferation marker gene. Therefore the observed up-regulation of *ZWINT* could be due to a higher proliferation index for the muscle-invasive tumors. *ZWINT* has indeed been shown to play a role in the mitotic checkpoint [48], [49], an essential step for cell division. Moreover, a previous study has shown that a high expression of *ZWINT* is associated with a poor prognosis in pulmonary carcinomas [50], thus the up-regulation of *ZWINT* could also be associated with a poor prognosis for bladder cancer. The fact that *ZWINT* is up-regulated in the muscle-invasive tumors but not in the superficial tumors is concordant with this hypothesis.

In addition to *RAB11A* and *ZWINT*, the other deregulated genes are also of interest. Their deregulation could be associated with events occurring before the establishment of bladder cancer or could be a consequence of genomic instability resulting from the malignant process. Among them *RAB23*, found to be up-regulated in this study, has been shown to be over-expressed and/or activated in hepatocellular carcinoma [51]. It was also found to be up-regulated in mucosa of atrophic gastritis and intestinal metaplasia [52] and it is required for the invasion of diffuse-type gastric cancer [9]. One RAB23 function is to antagonize sonic hedgehog (Shh)-mediated signalling during mouse development [53], a pathway implicated in urothelial cell proliferation in mice [54]. Of note, *RAB23* is highly expressed in muscle therefore, the up-regulation observed could be due to this stromal expression. Nevertheless it is also overexpressed in bladder cancer cell lines compared with normal urothelial cells grown in culture.

The down-regulation of *RAB4A*, encoding for a Rab that regulates fast recycling from early endosomes, and that of *CD2AP*, coding for a RAB4A effector involved in actin cytoskeleton regulation [55], points towards a general deregulation of the recycling pathway in T2–4 (*FGFR3*-non-mutated) tumors. The alteration of the fast recycling process was not found in the TaG1G2 (*FGFR3*-mutated) tumors. In these tumors *RAB4A* expression was not altered. The only RAB4A effector encoding gene whose expression was altered is *RAB11FIP1*, which is likely to be involved in urothelial cell differentiation (see above).

The possible role of the deregulation of intracellular trafficking of surface receptors in bladder cancer progression is supported by the finding that *RAB31* is up-regulated. RAB31 regulates EGFR trafficking in A431 cells [56]. Interestingly, EGFR is overexpressed in urothelial tumors [57]. Of note, high expression of *RAB31* is associated with a worse outcome in patients with breast cancer [58].

### Genes deregulated in both Ta G1G2 (*FGFR3*-mutated) and T2–4 (*FGFR3*-non-mutated) groups

Amongst this group of genes, *ITGA5* (up-regulated) encodes for the alpha 5 subunit of the alpha-5/beta-1 integrin that associates with RAB25 to promote invasive migration of ovarian carcinoma cells [59]. *ITGA5* is down-regulated in transformed plasma cells compared to normal plasma cells [60] and in highly invasive potential breast cancer cell lines [61]. Another study indicates, on the contrary, that *ITGA5* might be essential for breast cancer metastatic capacity [62]. We found an increase of *ITGA5* in tumors independently of the stage (stage Ta or stage T2–4) or of the pathway of bladder cancer progression. This suggests a role other than migration/invasion for *ITGA5* since Ta tumors do not have a high invasive potential.

### The Rab27 cluster

When the 284 genes analysed in this study were grouped in Rab-Rab effector clusters, we noticed that the Rab27 cluster was significantly deregulated in both pathways. In addition to *RAB27A/B*, five genes coding for RAB27 interacting partners (*GCC2*, *MLPH*, *RPH3AL*, *SYTL1* and *SYTL2*) were also down-regulated. In both pathways the deregulation of the Rab27 cluster is associated with the muscle-invasive tumors: *RAB27B*, *MLPH*, *RPH3AL* and *SYTL2* with the T2–4 (*FGFR3*-mutated) tumors and *GCC2*, *MLPH*, *RPH3AL*, *SYTL1* and *SYTL2* with the T2–4 (*FGFR3*-non-mutated) tumors. Interestingly, *MLPH* and *SYTL2* were found to be correlated with differentiation markers. *RAB27B*, even if it did not correlate with the urothelial differentiation markers used in this study, has been previously shown to be associated with fusiform vesicles in urothelial cells [63]. Therefore the association of the Rab27 cluster, of which all the genes were down-regulated, with muscle-invasive tumors could be linked to the loss of differentiation of muscle-invasive tumors. It is of interest to note that a recent study suggests that *RPH3AL* may have a role as a tumor suppressor gene in a minority of colorectal cancer cases [64].

### Proposed functions for some of the deregulated genes

Three genes were found to be linked with proliferation: *KIF20A, MYO5C* (inversely correlated) and *ZWINT* (discussed above). Finding *KIF20A* was not surprising, as this protein plays an essential role in cytokinesis [65], [66]. A previous study has also documented an up-regulation of *KIF20A* in bladder cancer [67]. This independent result aids in the validation of our approach. Finding *MYO5C* was more surprising since no previous study has linked this actin-based motor protein with proliferation. In addition, we could not confirm an association of *MYO5C* with proliferation using the NHU (normal human urothelial) cell culture model.

On the other hand, since bladder tumors tend to be less differentiated while they progress, we expected to find many down-regulated genes linked to differentiation. Seven genes: *ANKRD27*, *MLPH*, *MYO5B*, *RAB11A*, *RAB11FIP1*, *RAB20* and *SYTL2* among 30 down-regulated genes were indeed found to be positively correlated with differentiation markers. Therefore the majority of genes (23) found to be down-regulated in this study are not linked to differentiation. Interestingly, 6 of 9 genes linked to differentiation were found to be deregulated in the *FGFR3*-mutated group and not in the *FGFR3*-non-mutated group. In addition, this deregulation was already apparent in tumors of low stage and low grade (TaG1G2). Therefore, although TaG1G2 *FGFR3*-mutated tumors are well differentiated, they already present a loss of expression of several genes associated with differentiation. It remains to know whether these Rab genes are simply secondary markers of differentiation or if they play a role in bladder cancer pathogenesis. We expected to find more genes linked to differentiation being lost in the *FGFR3*-non-mutated tumor pathway as these tumors are usually of high grade compared to the tumors of the *FGFR3*-mutated tumor pathway. This was actually not the case. This unexpected result could be due to some heterogeneity in *FGFR3*-non-mutated tumors, as heterogeneity decreases the number of identified deregulated genes. A better characterization of bladder tumors should lead to the identification of new genes specifically deregulated in new subgroups of tumors.

In conclusion, the strategy applied in this study can be easily applied to any set of genes and any tumor subgroups. It requires biologists, specialists of a given pathway/process, to select the most appropriate set of genes and to interpret the results. In many tumors, the normal counterpart of the transformed cell is not available or not known. In this case, one can compare tumors of different stages and grades. The next step will be to address the cellular functions of the genes deregulated at the transcriptome level and their possible implication in cancer pathogenesis.

## Materials and Methods

### Establishment of the list of genes

We first used the GeneCards database and found 61 human *RAB* genes indexed. Then using the *RAB* gene name and the different aliases listed as key search words we found in 185 articles indexed in PubMed a total of 217 proteins whose interaction with one or several Rab proteins has been published up to April 2010. After adding *GDI1*, *GDI2*, *CHM*, *CHML*, *RABGGTA* and *RABGGTB*, we worked with a total of 284 genes. We adopted HUGO gene nomenclature, except for *CP110* since its Entrez Gene name had not been indexed in the HUGO database.

### Normal and tumor samples

Tumors were staged according to the TNM classification [68] and graded according to the criteria recommended by the World Health Organization (1973 classification) [69]. *FGFR3* mutation was assessed by direct sequencing or by the SNaPshot method [70].

Two sets of independent tumors obtained during transurethral resection or radical cystectomy [71] and normal urothelium samples obtained from organ donors [72] were used. The tumors were provided by Drs A.-C. Baglin and Y. Denoux (Tumorothèque, Service d'Anatomie et Cytologie Pathologiques, CMC Foch, Suresnes, France), Dr M.-J. Terrier-Lacombe (Service de Pathologie, Institut Gustave Roussy, Villejuif, France), and Pr K. Leroy (Plateforme de Ressources Biologiques, Pôle de Recherche Clinique, APHP, Hôpital Henri Mondor, Créteil, France).

The first set is composed of 4 isolated samples of normal urothelium and 152 tumor samples: 28 TaG1G2 (*FGFR3*-mutated), 2 TaG3 (*FGFR3*-mutated), 13 T1 (*FGFR3*-mutated), 9 T2–4 (*FGFR3*-mutated), 9 TaG1G2 (*FGFR3*-non-mutated), 3 TaG3 (*FGFR3*-non-mutated), 25 T1 (*FGFR3*-non-mutated) and 63 T2–4 (*FGFR3*-non-mutated). These samples were analyzed with Affymetrix HG U133 Plus 2.0 DNA microarrays. 269 of the 284 genes listed were found in these microarrays when using BrainArray Annotation ENTREZGENE [73]. *F8A1*, *CCZ1*, *GMCL1*, *RAB13*, *RAB19*, *RAB41*, *RAB43*, *RAB44*, *RAB6C*, *RAB7B*, *RAB9B*, *REP15*, *RPH3A*, *TBC1D3B* and *YWHAQ* were absent.

The second set is composed of 5 isolated samples of normal urothelium and 75 tumor samples: 18 TaG1G2 (*FGFR3*-mutated), 1 TaG3 (*FGFR3*-mutated), 5 T1 (*FGFR3*-mutated), 7 T2–4 (*FGFR3*-mutated), 3 TaG3 (*FGFR3*-non-mutated), 2 TaG1G2 (*FGFR3*-non-mutated), 7 T1 (*FGFR3*-non-mutated) and 32 T2–4 (*FGFR3*-non-mutated). These samples were analyzed with Affymetrix HG U95A/U95Av2 DNA microarrays. 165 of the 284 genes listed were present in custom chip definition of BrainArray ENTREZGENE.

We worked with 7 bladder cancer cell lines: KK47, MGHU3, RT112, RT4, SCaBER, SD48 and T24, and normal human urothelial (NHU) cells grown in culture following previous protocols [27], [28]. These cell lines were analyzed with Affymetrix Human exon 1.0stV2 microarrays. 281 of the 284 genes listed were present in remapped chip definition: CCZ1, F8A1 and RAB7B were absent. RT4, RT112 and T24 were obtained from DSMZ (Braunschweig, Germany), SCaBER from ATCC (Rockville, MD), KK47 and SD48 from the laboratory of D. Chopin (Hôpital Henri Mondor, Créteil, France) and MGHU3 from the laboratory of Y. Fradet (University of Laval, Québec, Canada). To verify the identity of the various cell lines used, we analyzed the genomic alterations with comparative genomic hybridization (CGH) arrays and FGFR3, TP53, HRAS, and KRAS mutations with the SNaPshot technique (for FGFR3) or classical sequencing for the other genes. CGH array profiles were found to be similar to published CGH profiles and/or consistent with previously reported genomic alterations, and mutations were found to be identical to the reported mutations (data not shown).

The NHU cell cultures used for the last part of the results were established and maintained in culture as described [27] and were induced to differentiate by treatment with an EGFR (epidermal growth factor receptor) inhibitor and an activator of PPARγ (peroxisome proliferator-activated receptor gamma) [28], [74]. Differentiated and untreated cultures were analyzed with Affymetrix HG U133 Plus 2.0 DNA microarrays.

RNA was prepared from the normal and tumor samples and cancer cell lines with the cesium chloride procedure [75].

All patients provided written informed consent and the study was approved by the ethics committees of the different hospitals (Comité de Protection des Personnes de l'hôpital Henri Mondor, Comité de Protection des Personnes de Boulogne – Ambroise Paré and Comité de Protection des Personnes de Bicêtre). All analyses were performed on the basis of anonymized patient data.

### Affymetrix microarray data

We used the Human Genome U95A, U95Av2, U133Plus2.0 and Human Exon 1.0st V2 arrays (Affymetrix). Details of the methods for RNA amplification, cDNA probe labeling and hybridization steps can be obtained from the Affymetrix web site. All gene expression data were normalized and summarized using RMA (Robust Multi-array Averaging) algorithm [76] with custom chip definition developed by Microarray Lab, BrainArray [73].

BrainArray annotation ENTREGENE (version 12, available at http://brainarray.mbni.med.umich.edu/Brainarray/Database/CustomCDF/CDF_download.asp#v12) providing one remapped probe set per gene according to National Center for Biotechnology Information (NCBI) *Homo sapiens* ENTREGENE build 36.1 was used. One log2-transformed signal value per gene was obtained. The all normalized microarray data sets are available online at http://microarrays.curie.fr/publications/UMR144/RabBladder/ (login: rabbld; password: 248ba64). Concerning the deposition in ArrayExpress, we do not expect to receive the accession numbers immediately and will notify PLoS ONE when we will receive them.

Data were generated by using R language environment (version 2.12.0, available at http://www.r-project.org/) and bioconductor packages (available at http://www.bioconductor.org/).

### Method of analysis

We used the statistical SAM (Significance Analysis of Microarrays) test [77], adapted for Excel, to compare the transcriptional levels of the genes between two different groups. We used Log_2_ (data) and the following parameters: Two class unpaired; Analysis type (Standard (genes)); T test statistic; No median center the array; Number of permutations: 100; Automatic estimate for s0 factor for denominator; Imputation Engine: K-Nearest Neighbours Imputer; Number of Neighbours: 10; Random number seed: 1234567.

We used Microsoft Office Excel 2003 to calculate the Pearson correlation. Log_2_(data) was used. The significance (two-tailed probability) of the Pearson correlation was calculated using the p-Value calculator of danielsoper.com (http://www.danielsoper.com/statcalc/calc44.aspx).

The binomial test on R (binom.test) was used with the parameter: alternative = c(“greater”).

The Student test pValue was calculated with the following Excel parameters: two tails and different variance hypothesis.

## Supporting Information

Figure S1 **Rab clusters.** A Rab cluster is defined as a Rab and its interacting proteins: the GEFs (guanine nucleotide exchange factors), the GAPs (GTPase activating proteins) and the effector proteins. Rab proteins are in yellow, GEFs in green, GAPs in light red and effector proteins in blue.
(PDF)

Table S1 **List of papers in which the Rab partners were originally described.**
(XLS)

Table S2 **Genes deregulated during bladder cancer pathogenesis: second set of data.** Second set of data. Affymetrix HG U95A/U95Av2 DNA microarrays. Genes down- or up-regulated in the tumor samples. Left: FGFR3-non-mutated tumor groups. Right: *FGFR3*-mutated tumor groups. The results shown to pass the thresholds: FC>1.5 (or <0.667) and qValue <5% with the first set of data are in red (or green) or highlighted in red (or green). The highlighted values in red or green pass the thresholds FC>1.5 or FC <0.667 or qValue <5% with the second set of data also. The results highlighted in yellow pass the thresholds: FC>1.5 (or <0.667) and qValue <5% only with the second set of data.
(PDF)

Table S3 **Genes deregulated only in** ***FGFR-*** **non-mutated (A) and mutated tumors (B).** Genes up- (or down)-regulated that pass the thresholds: FC>1.5 (or <0.667) and qValue <5% only for the *FGFR3-*non-mutated (A) or *FGFR3-*mutated (B) tumor pathway. Left column: comparison with the normal urothelium samples. Right column: comparison with the tumor samples of same stage with the opposite *FGFR3* mutation status. The results that pass the above thresholds are highlighted (red for up-regulation and green for down-regulation).
(PDF)

Table S4 **Genes significantly up- or down-regulated in each Rab cluster for each tumor group of the two pathways.** “D” (green) corresponds to a gene significantly down-regulated in the tumor samples, “U” (red) corresponds to a gene significantly up-regulated in the tumor samples. “% All genes” corresponds to the total number of genes up- or down-regulated within each tumor group. A binomial test was applied for each Rab cluster within each tumor group. The binomial test pValues <1% are highlighted in orange. “Absent” corresponds to a gene absent in the Affymetrix HG U133 Plus 2.0 DNA microarrays.
(XLS)

Table S5 **Pearson correlation (r) (and pValue) between the expression of** ***MKI67*** **and the expression of genes listed in left column.** Pearson correlation (r) (and pValue) between the expression of *MKI67* and the expression of genes listed in left column in 28 Ta G1G2 (*FGFR3*-mutated) tumor samples and 63 T2-4 (*FGFR3*-non-mutated) tumor samples. Are highlighted (in green or red) when |r| >0.479 for the Ta G1G2 (*FGFR3*-mutated) group (28 samples) and |r| >0.323 for the T2–4 (*FGFR3*-non-mutated) group (63 samples).
(PDF)

Table S6 **Pearson correlation (r) (and pValue) between the expression of different urothelial differentiation markers genes and the expression of genes listed in left column.** Pearson correlation (r) (and pValue) between the expression of *FOXA1*, *GRHL3*, *UPK1A/B*, *UPK2*, *UPK3A/B* and the expression of genes listed in left column in 28 Ta G1/G2 (*FGFR3*-mutated) tumor samples and 63 T2–4 (*FGFR3*-non-mutated) tumor samples. Are highlighted (red corresponds to a correlation, green corresponds to an inverse correlation) when the two correlation values pass the thresholds: |r| >0.479 (pValue <1%) for the Ta G1G2 (*FGFR3*-mutated) group (28 samples) and |r| >0.323 (pValue <1%) for the T2–4 (*FGFR3*-non-mutated) group (63 samples).
(XLS)
