# Supplementary material for: Deregulation of Rab and Rab Effector Genes in Bladder Cancer
Source: PLoS One. 2012 Jun 19;7(6):e39469. doi: 10.1371/journal.pone.0039469 (PMC3378553; doi:10.1371/journal.pone.0039469)
Supplement: Figure S1 — Rab clusters. A Rab cluster is defined as a Rab and its interacting proteins: the GEFs (guanine nucleotide exchange factors), the GAPs (GTPase activating proteins) and the effector proteins. Rab proteins are in yellow, GEFs in green, GAPs in light red and effector proteins in blue. (PDF) [file pone.0039469.s001.pdf]

Figure S1.1

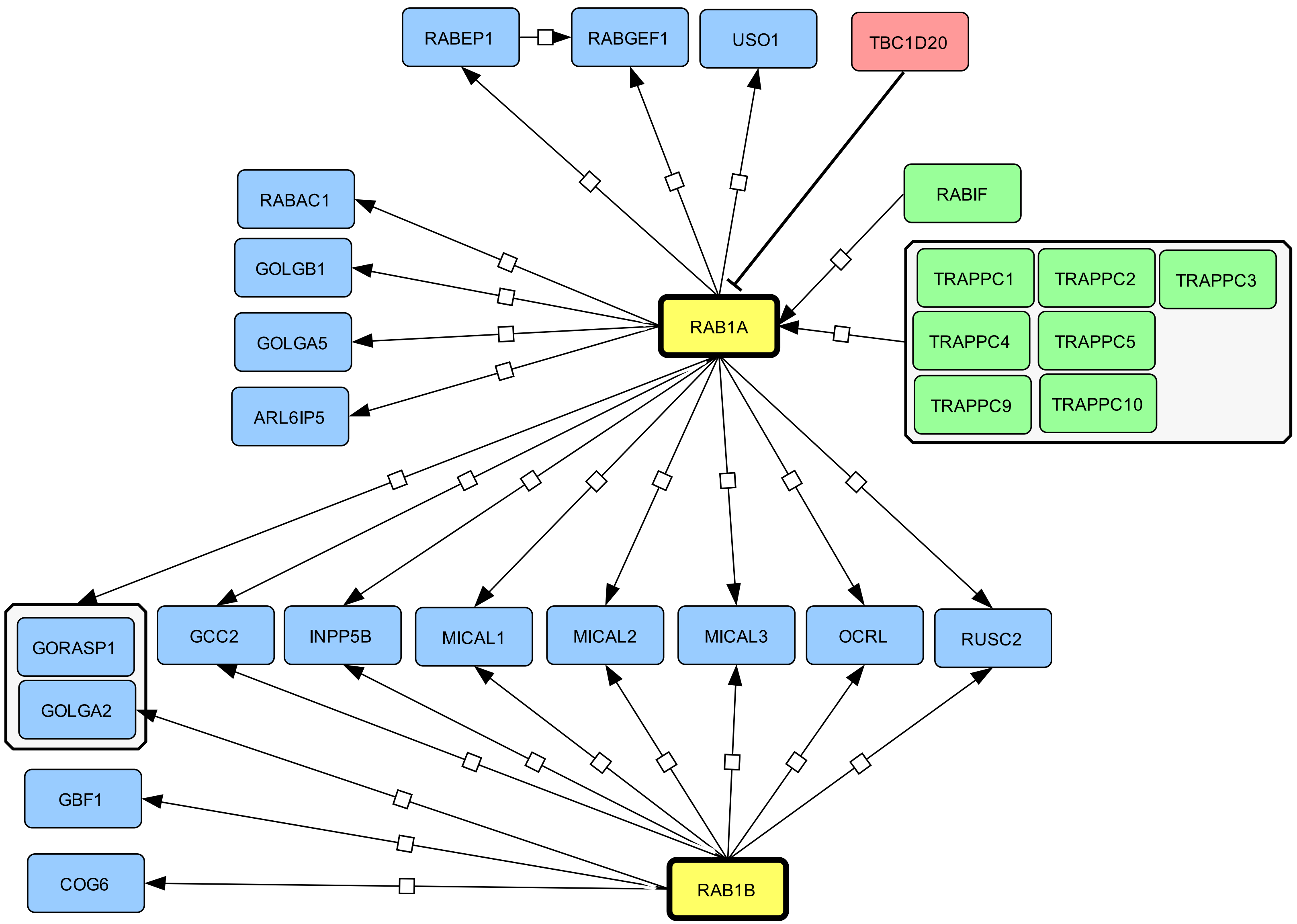

RABEP1
RABGEF1
USO1
TBC1D20
RABIF
RABAC1
GOLGB1
GOLGA5
ARL6IP5
RAB1A
TRAPPC1
TRAPPC2
TRAPPC3
TRAPPC4
TRAPPC5
TRAPPC9
TRAPPC10
GORASP1
GOLGA2
GCC2
INPP5B
MICAL1
MICAL2
MICAL3
OCRL
RUSC2
GBF1
COG6
RAB1B

Figure S1.2

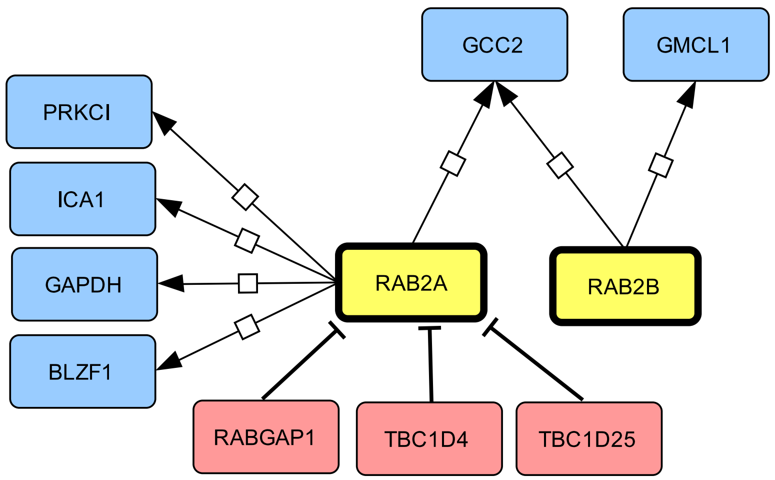
GCC2
GMCL1
PRKCI
ICA1
GAPDH
BLZF1
RAB2A
RAB2B
RABGAP1
TBC1D4
TBC1D25

Figure S1.3

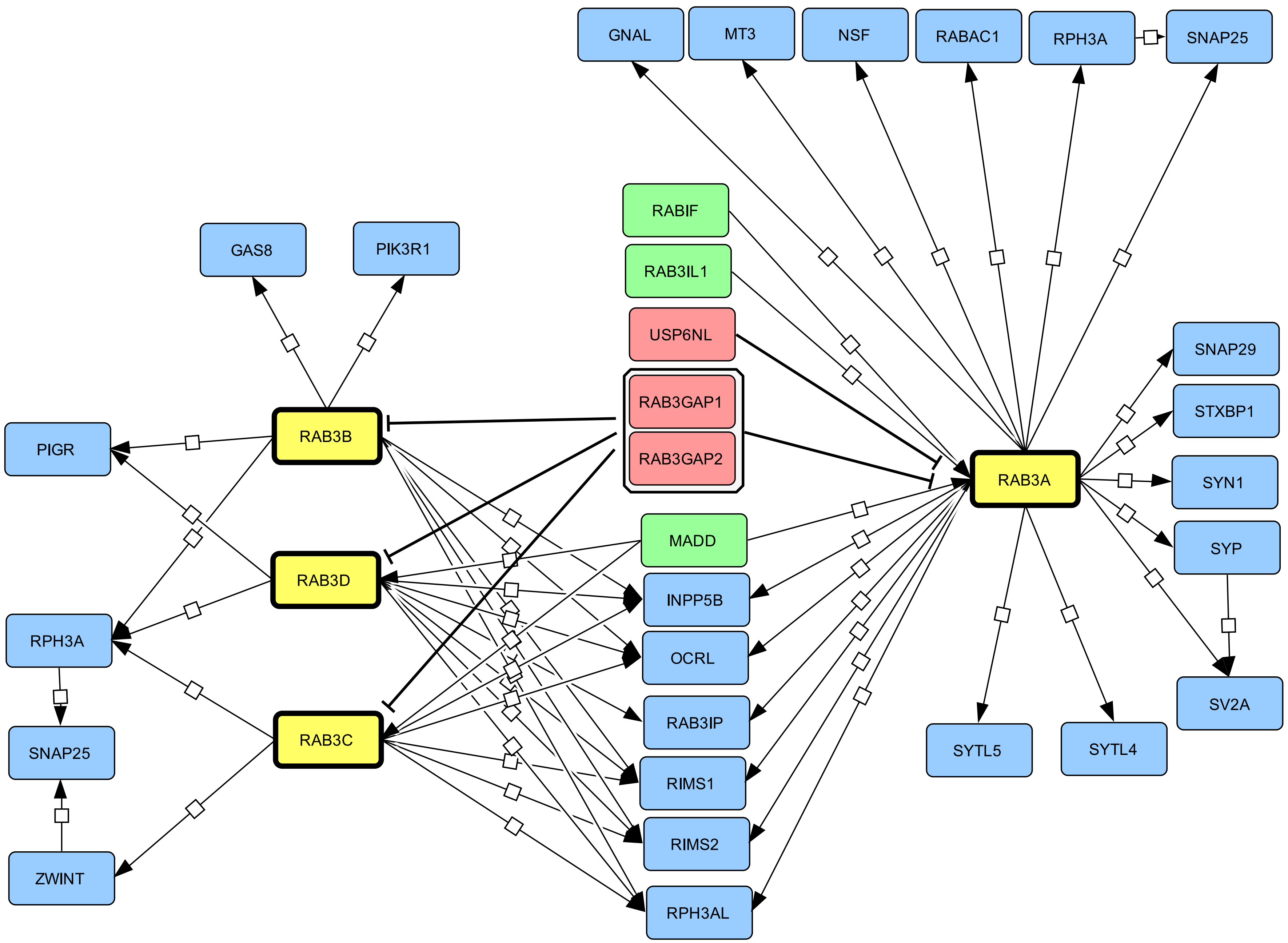

GNAL
MT3
NSF
RABAC1
RPH3A
SNAP25
RABIF
RAB3IL1
USP6NL
RAB3GAP1
RAB3GAP2
MADD
INPP5B
OCRL
RAB3IP
RIMS1
RIMS2
RPH3AL
RAB3A
SNAP29
STXBP1
SYN1
SYP
SV2A
SYTL5
SYTL4
GAS8
PIK3R1
RAB3B
RAB3D
RAB3C
PIGR
RPH3A
SNAP25
ZWINT

Figure S1.4

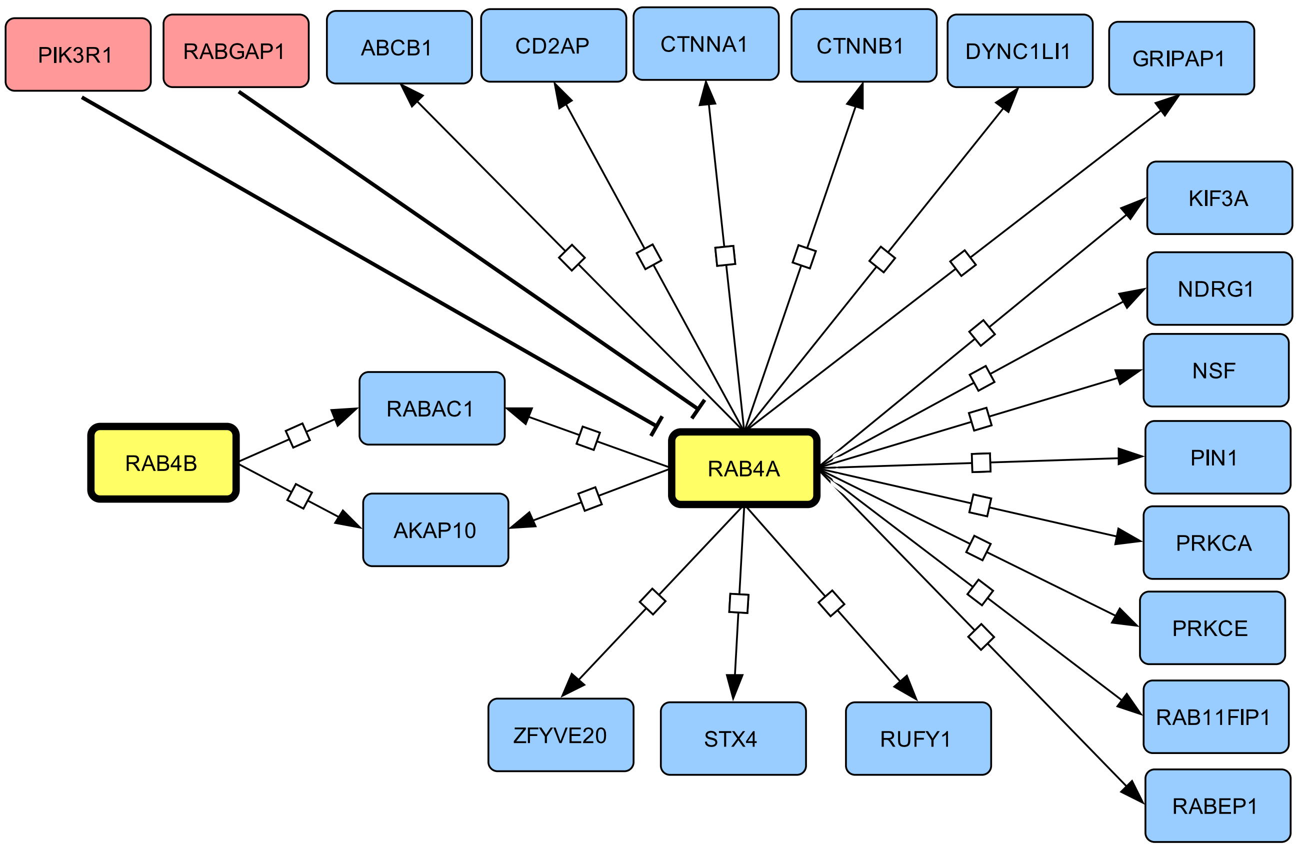

PIK3R1
RABGAP1
ABCB1
CD2AP
CTNNA1
CTNNB1
DYNC1LI1
GRIPAP1
KIF3A
NDRG1
NSF
PIN1
PRKCA
PRKCE
RAB11FIP1
RABEP1
RAB4B
RABAC1
AKAP10
RAB4A
ZFYVE20
STX4
RUFY1

Figure S1.5

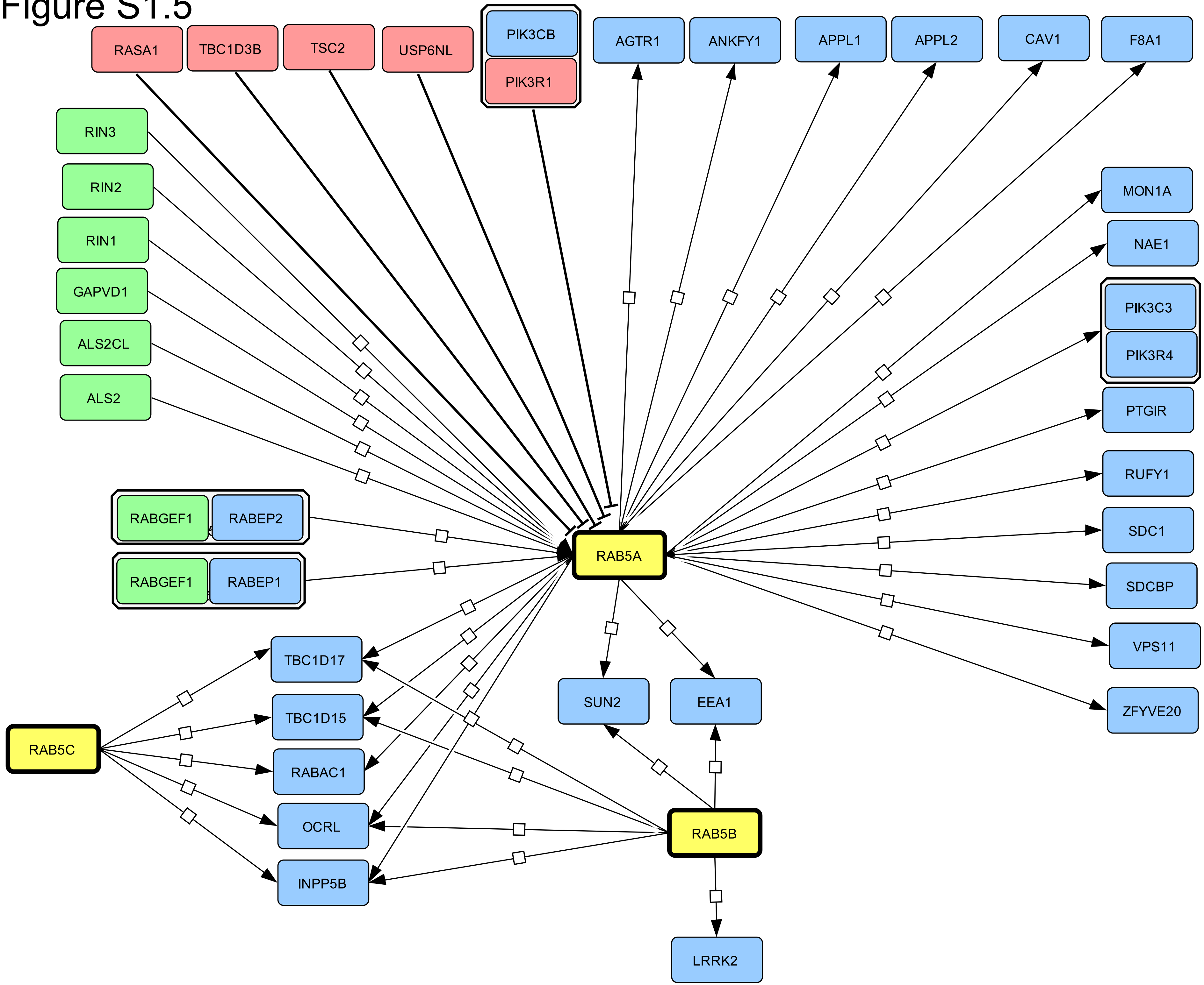

RASA1
TBC1D3B
TSC2
USP6NL
PIK3CB
PIK3R1
AGTR1
ANKFY1
APPL1
APPL2
CAV1
F8A1
RIN3
RIN2
RIN1
GAPVD1
ALS2CL
ALS2
MON1A
NAE1
PIK3C3
PIK3R4
PTGIR
RUFY1
SDC1
SDCBP
VPS11
ZFYVE20
RABGEF1
RABEP2
RABGEF1
RABEP1
RAB5A
TBC1D17
TBC1D15
RABAC1
OCRL
INPP5B
RAB5C
SUN2
EEA1
RAB5B
LRRK2

Figure S1.6

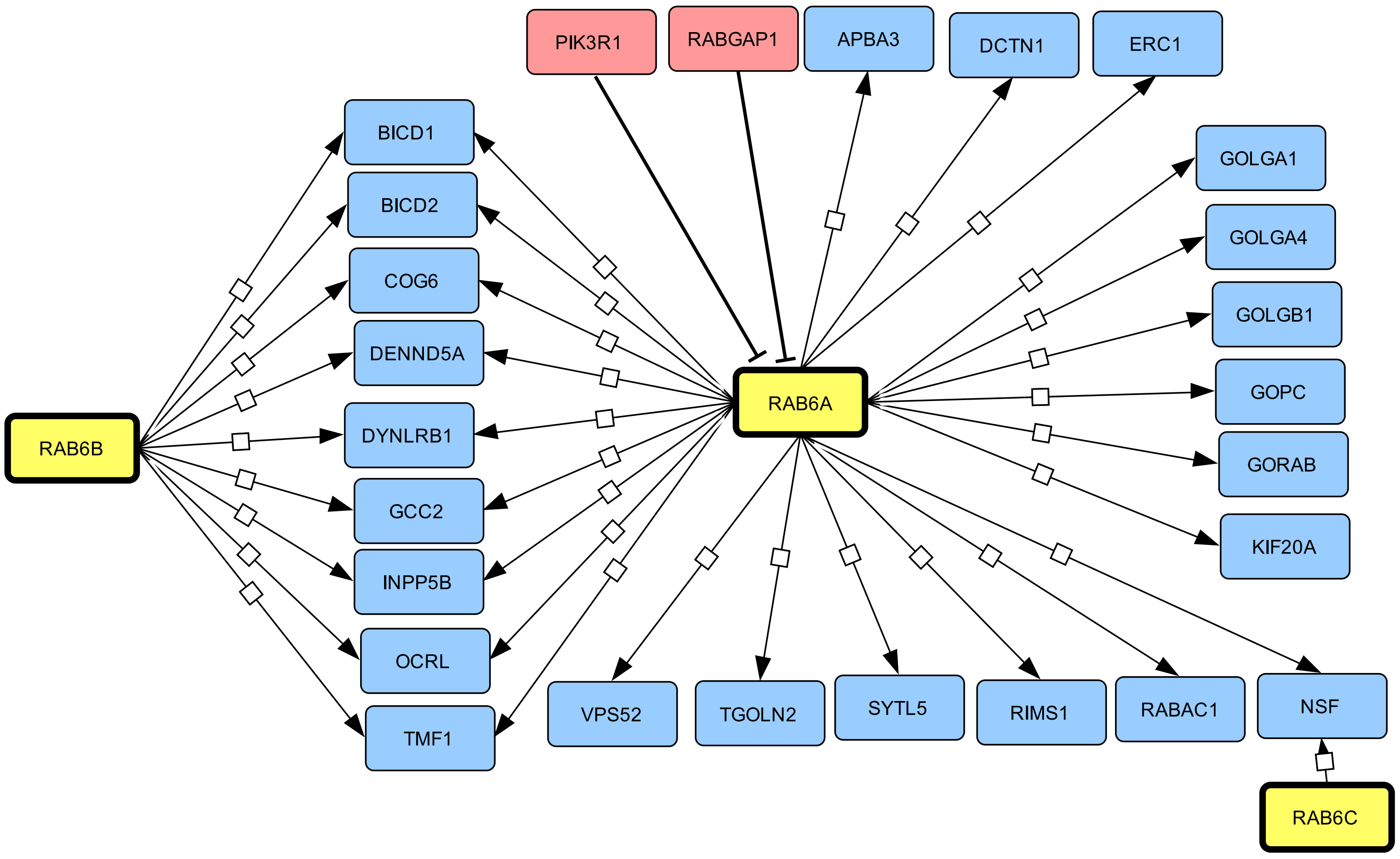

PIK3R1
RABGAP1
APBA3
DCTN1
ERC1
BICD1
BICD2
COG6
DENND5A
DYNLRB1
GCC2
INPP5B
OCRL
TMF1
RAB6B
RAB6A
GOLGA1
GOLGA4
GOLGB1
GOPC
GORAB
KIF20A
VPS52
TGOLN2
SYTL5
RIMS1
RABAC1
NSF
RAB6C

Figure S1.7

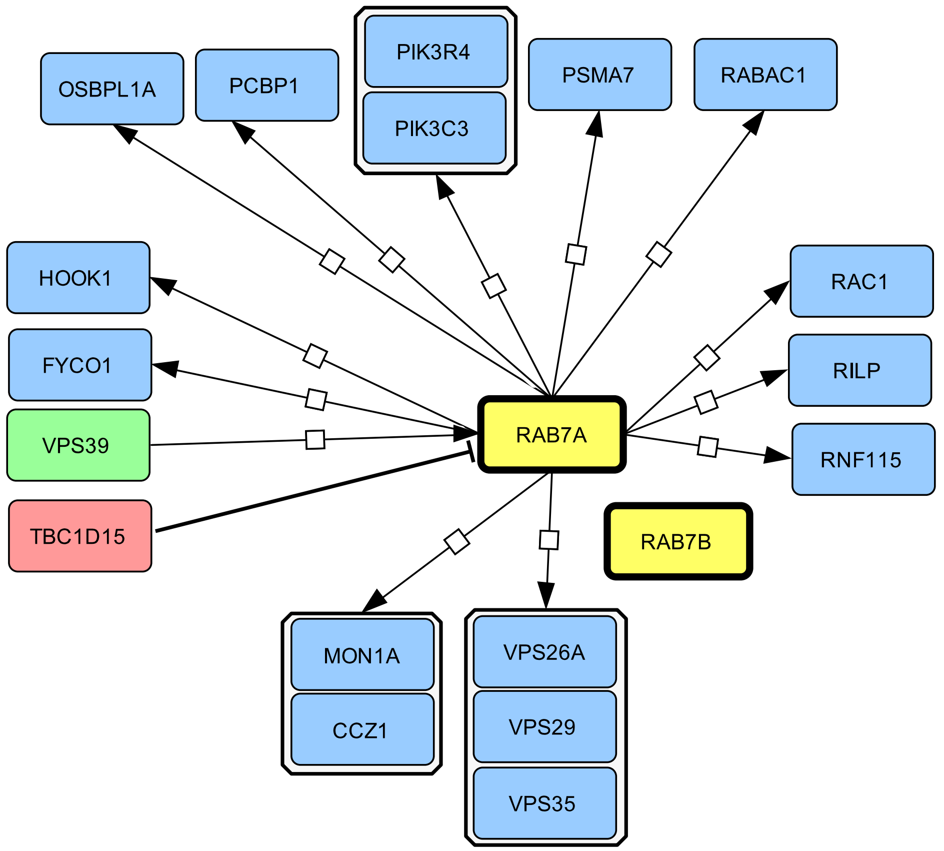
OSBPL1A
PCBP1
PIK3R4
PIK3C3
PSMA7
RABAC1
HOOK1
FYCO1
VPS39
TBC1D15
RAB7A
RAC1
RILP
RNF115
RAB7B
MON1A
CCZ1
VPS26A
VPS29
VPS35

Figure S1.8

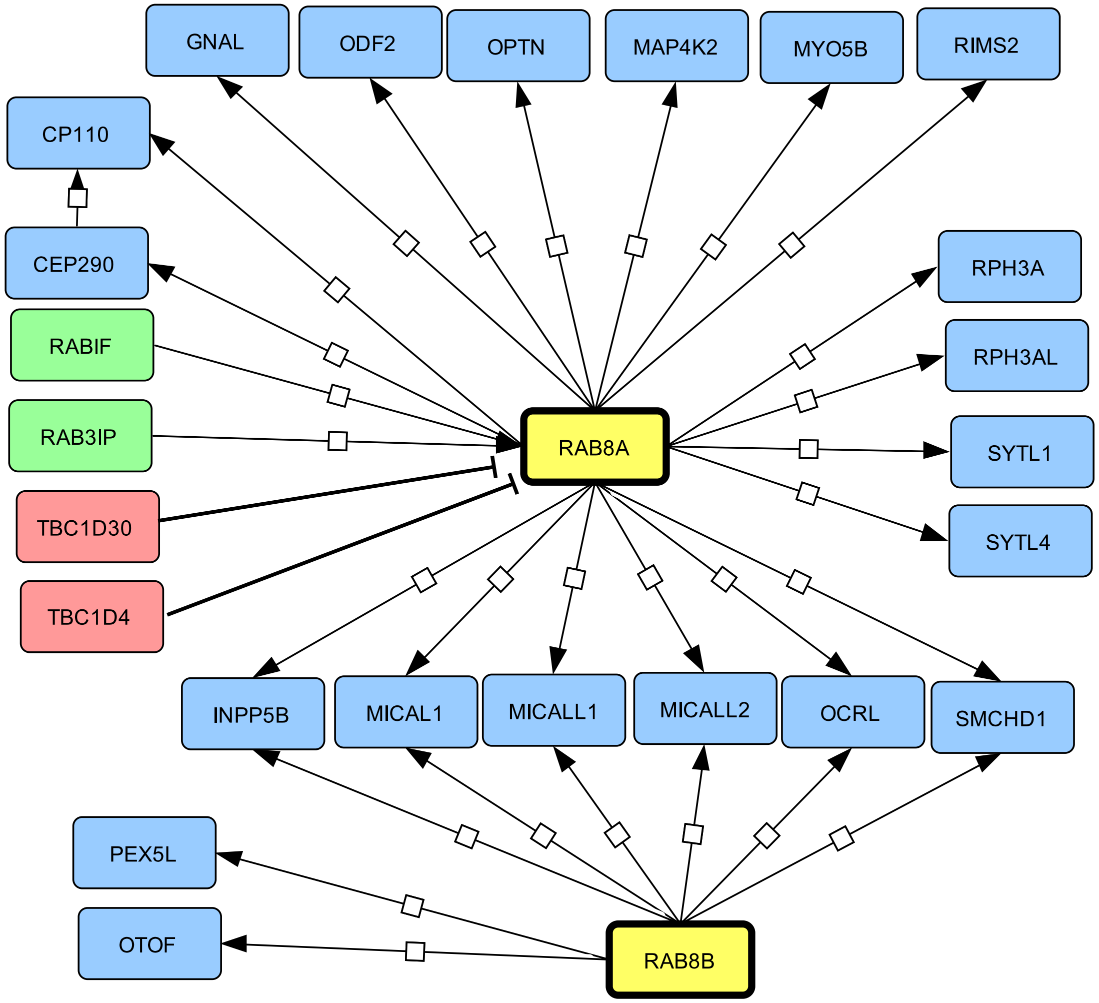
GNAL
ODF2
OPTN
MAP4K2
MYO5B
RIMS2
CP110
CEP290
RABIF
RAB3IP
TBC1D30
TBC1D4
RAB8A
RPH3A
RPH3AL
SYTL1
SYTL4
INPP5B
MICAL1
MICALL1
MICALL2
OCRL
SMCHD1
PEX5L
OTOF
RAB8B

Figure S1.9

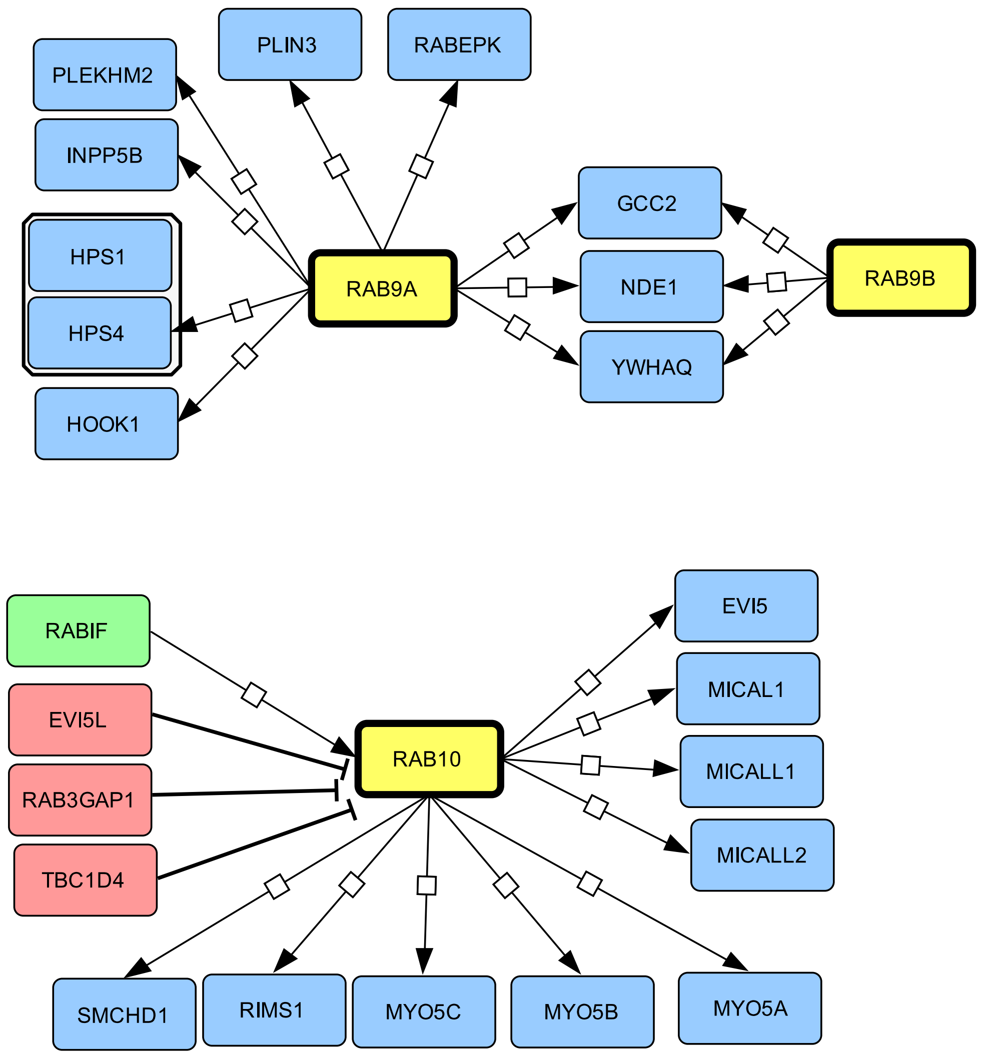
PLEKHM2
PLIN3
RABEPK
INPP5B
HPS1
HPS4
HOOK1
RAB9A
GCC2
NDE1
YWHAQ
RAB9B
RABIF
EVI5L
RAB3GAP1
TBC1D4
RAB10
EVI5
MICAL1
MICALL1
MICALL2
SMCHD1
RIMS1
MYO5C
MYO5B
MYO5A

Figure S1.10

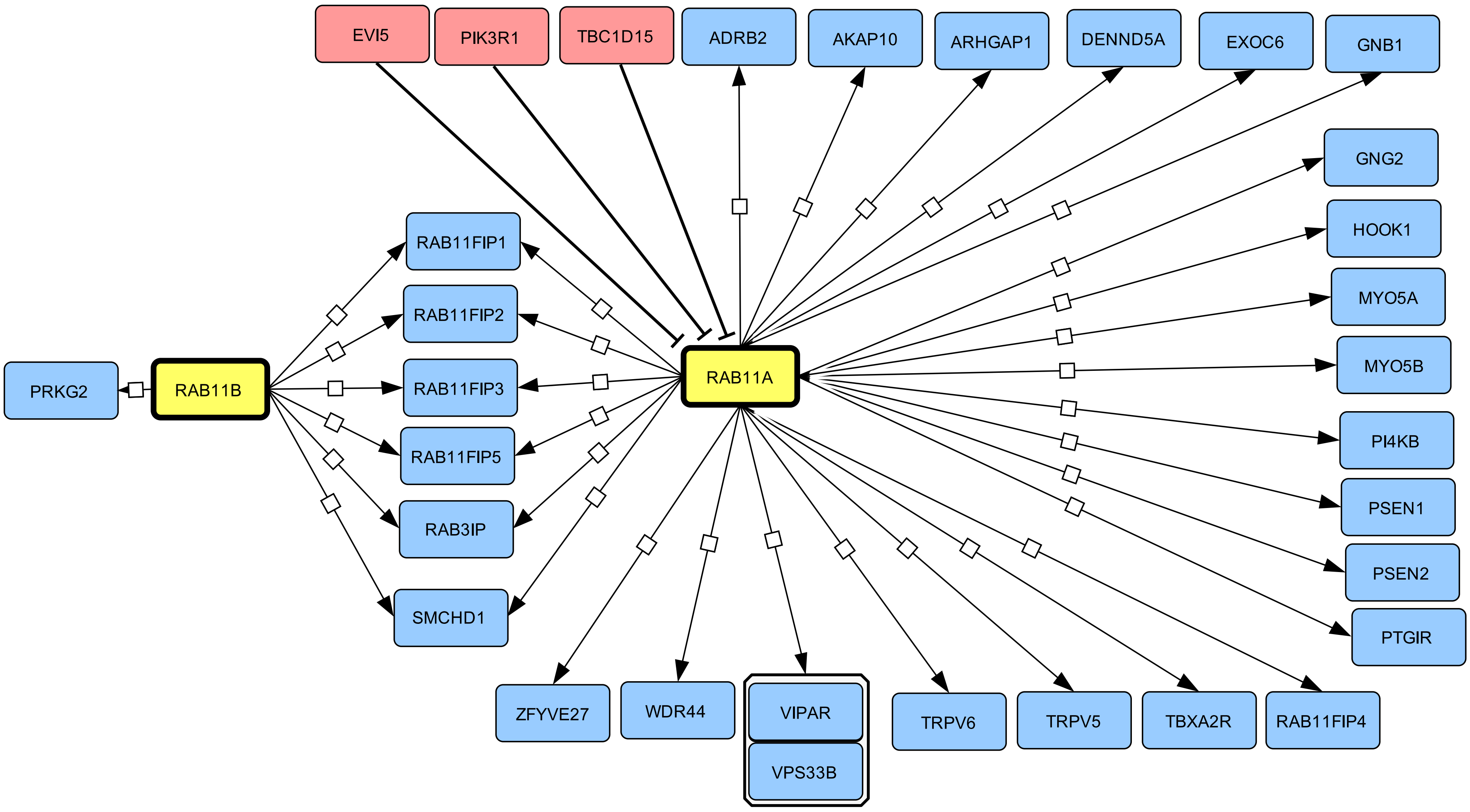
EVI5
PIK3R1
TBC1D15
ADRB2
AKAP10
ARHGAP1
DENND5A
EXOC6
GNB1
GNG2
HOOK1
MYO5A
MYO5B
PI4KB
PSEN1
PSEN2
PTGIR
PRKG2
RAB11B
RAB11A
RAB11FIP1
RAB11FIP2
RAB11FIP3
RAB11FIP5
RAB3IP
SMCHD1
ZFYVE27
WDR44
VIPAR
VPS33B
TRPV6
TRPV5
TBXA2R
RAB11FIP4

Figure S1.11

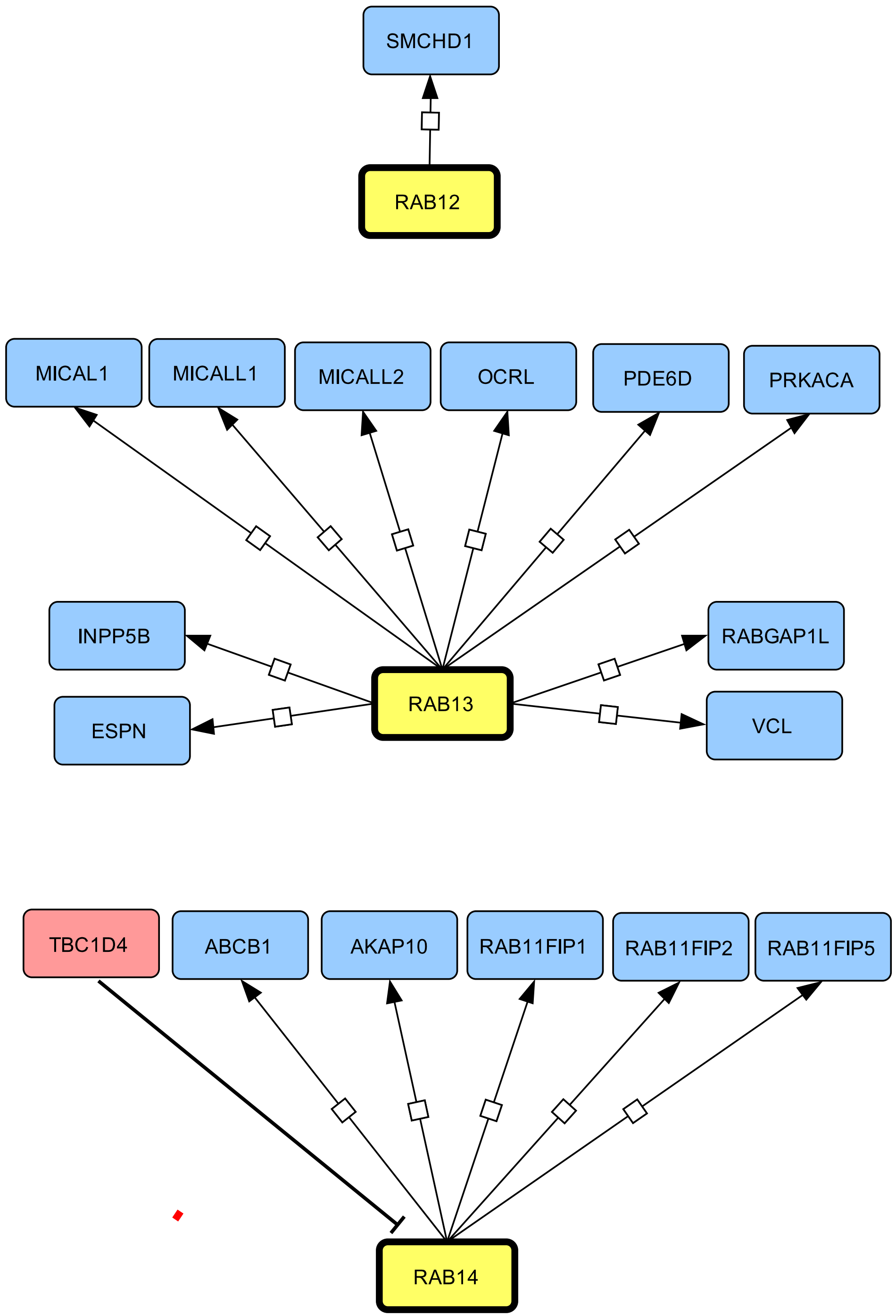

SMCHD1
RAB12
MICAL1
MICALL1
MICALL2
OCRL
PDE6D
PRKACA
INPP5B
RABGAP1L
ESPN
RAB13
VCL
TBC1D4
ABCB1
AKAP10
RAB11FIP1
RAB11FIP2
RAB11FIP5
RAB14

Figure S1.12

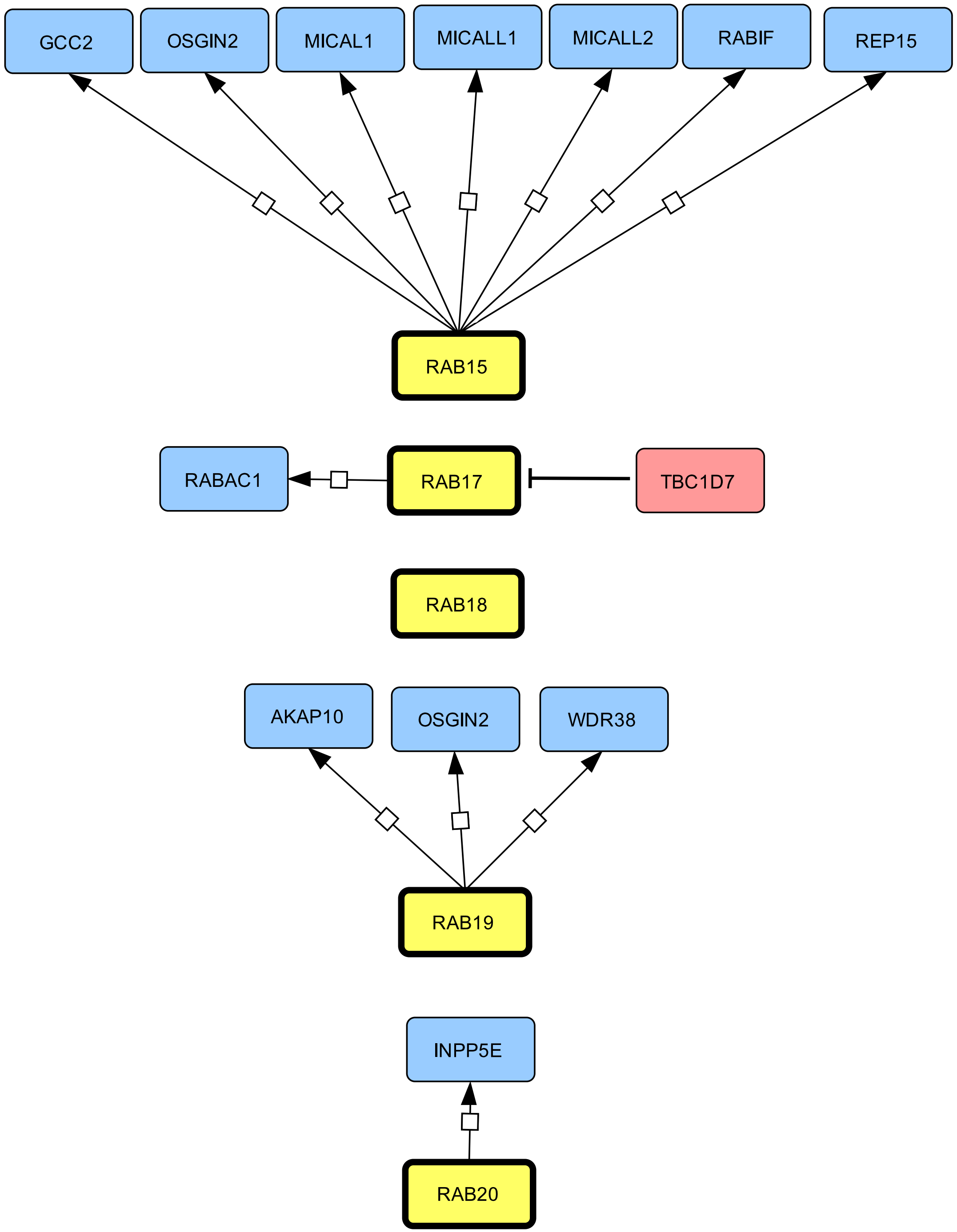

GCC2
OSGIN2
MICAL1
MICALL1
MICALL2
RABIF
REP15
RAB15
RABAC1
RAB17
TBC1D7
RAB18
AKAP10
OSGIN2
WDR38
RAB19
INPP5E
RAB20

Figure S1.13

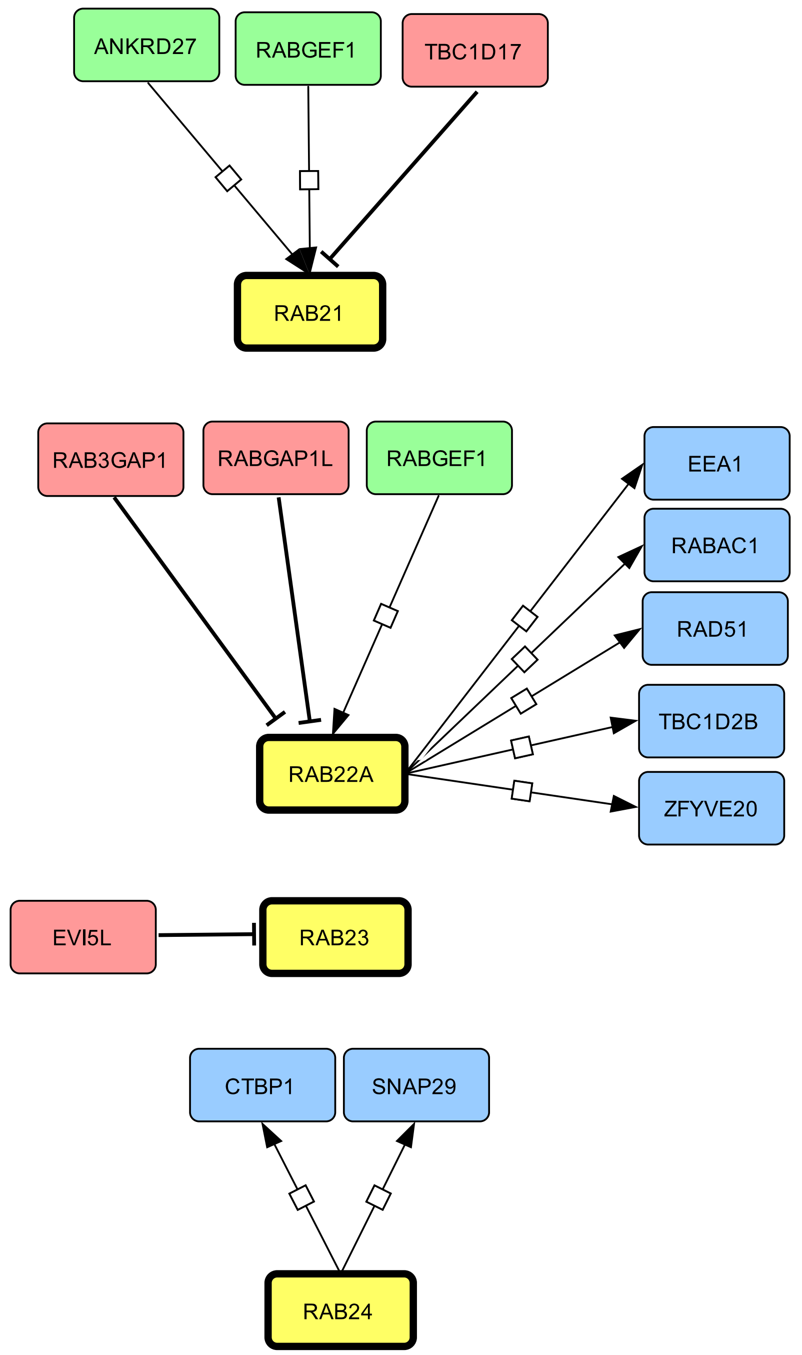
ANKRD27
RABGEF1
TBC1D17
RAB21
RAB3GAP1
RABGAP1L
RABGEF1
EEA1
RABAC1
RAD51
TBC1D2B
ZFYVE20
RAB22A
EVI5L
RAB23
CTBP1
SNAP29
RAB24

Figure S1.14

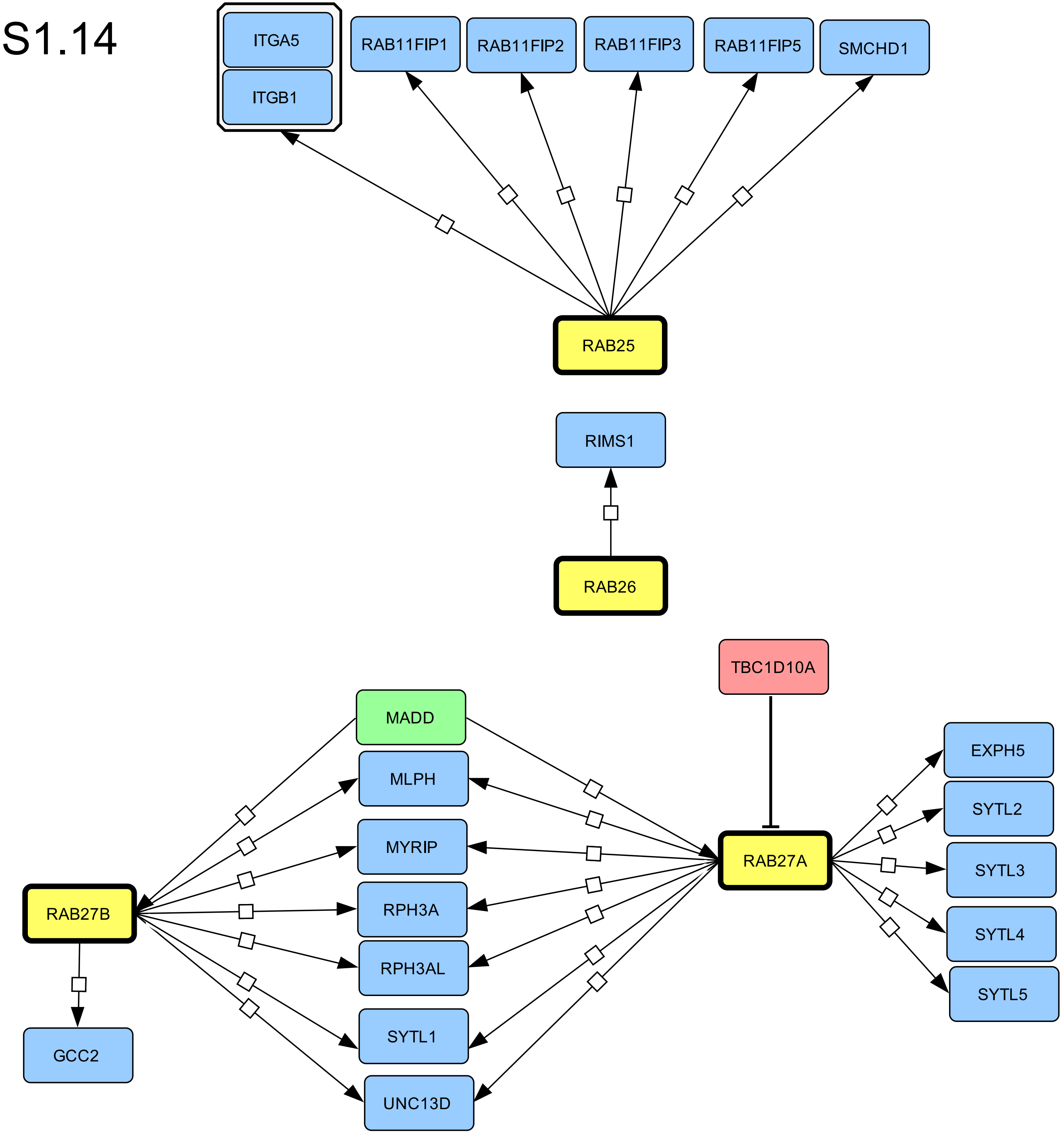

ITGA5
ITGB1
RAB11FIP1
RAB11FIP2
RAB11FIP3
RAB11FIP5
SMCHD1
RAB25
RIMS1
RAB26
TBC1D10A
MADD
MLPH
MYRIP
RPH3A
RPH3AL
SYTL1
UNC13D
RAB27B
GCC2
RAB27A
EXPH5
SYTL2
SYTL3
SYTL4
SYTL5

Figure S1.15

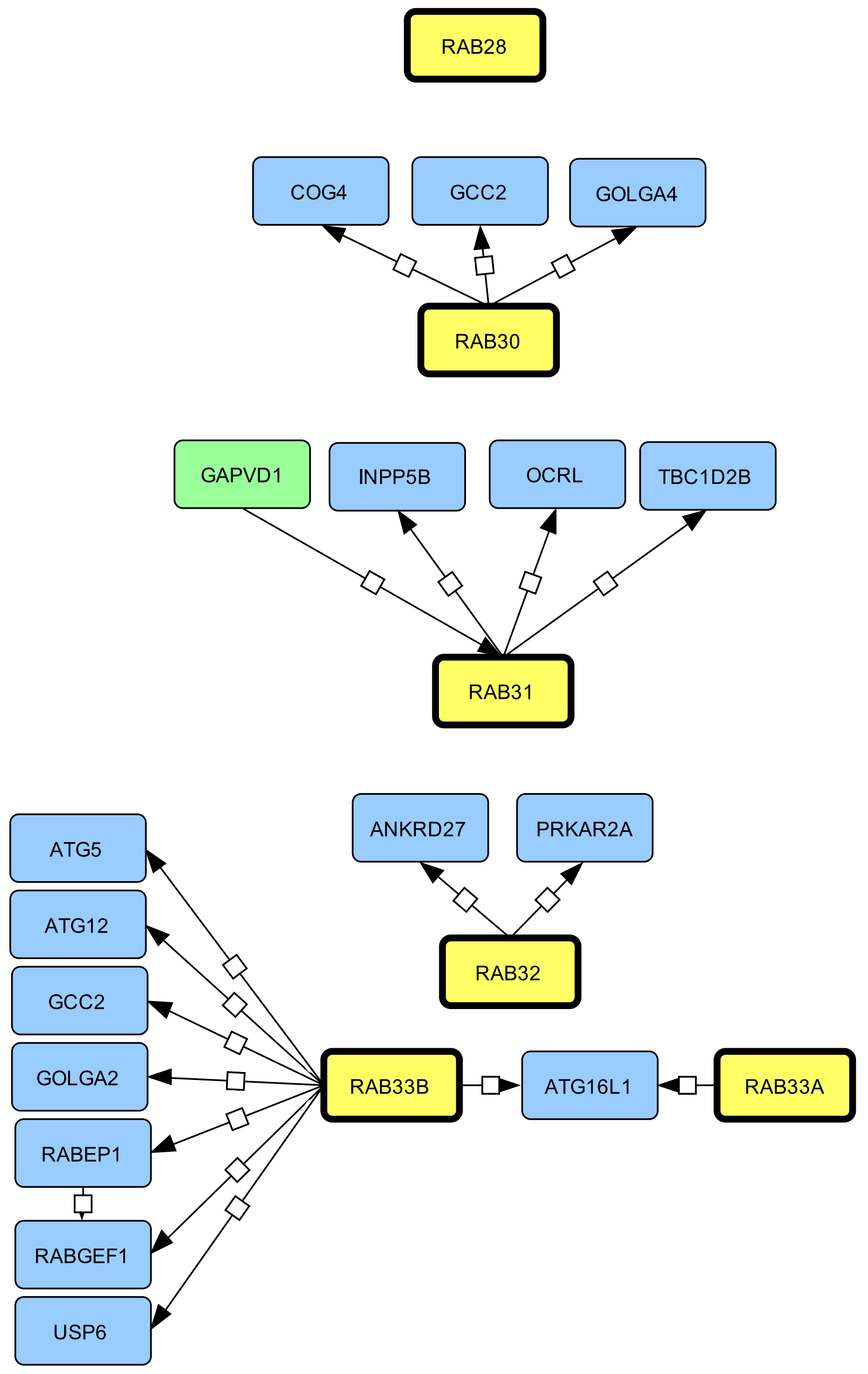
RAB28
COG4
GCC2
GOLGA4
RAB30
GAPVD1
INPP5B
OCRL
TBC1D2B
RAB31
ANKRD27
PRKAR2A
RAB32
ATG5
ATG12
GCC2
GOLGA2
RABEP1
RABGEF1
USP6
RAB33B
ATG16L1
RAB33A

Figure S1.16

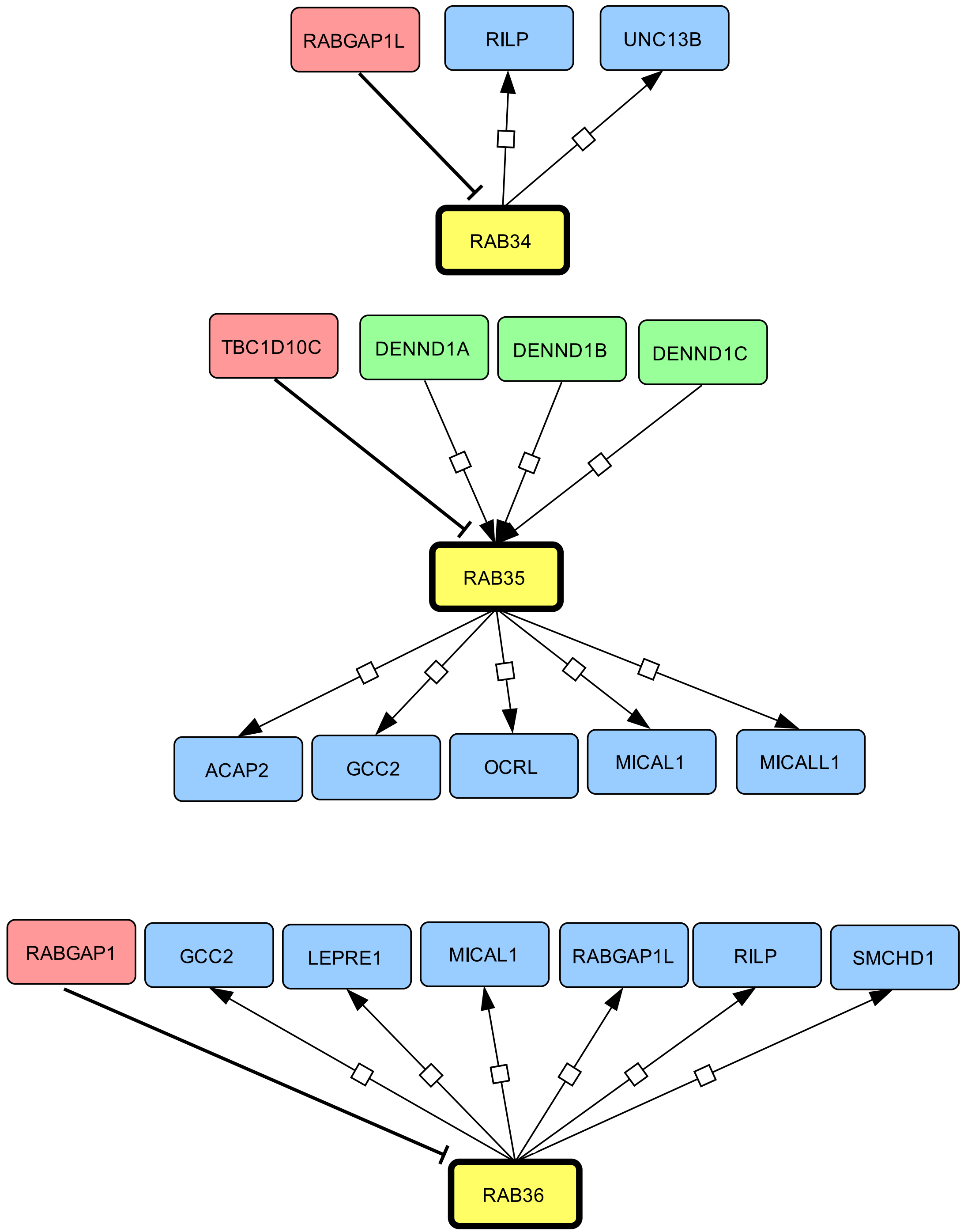
RABGAP1L
RILP
UNC13B
RAB34
TBC1D10C
DENND1A
DENND1B
DENND1C
RAB35
ACAP2
GCC2
OCRL
MICAL1
MICALL1
RABGAP1
GCC2
LEPRE1
MICAL1
RABGAP1L
RILP
SMCHD1
RAB36

Figure S1.17

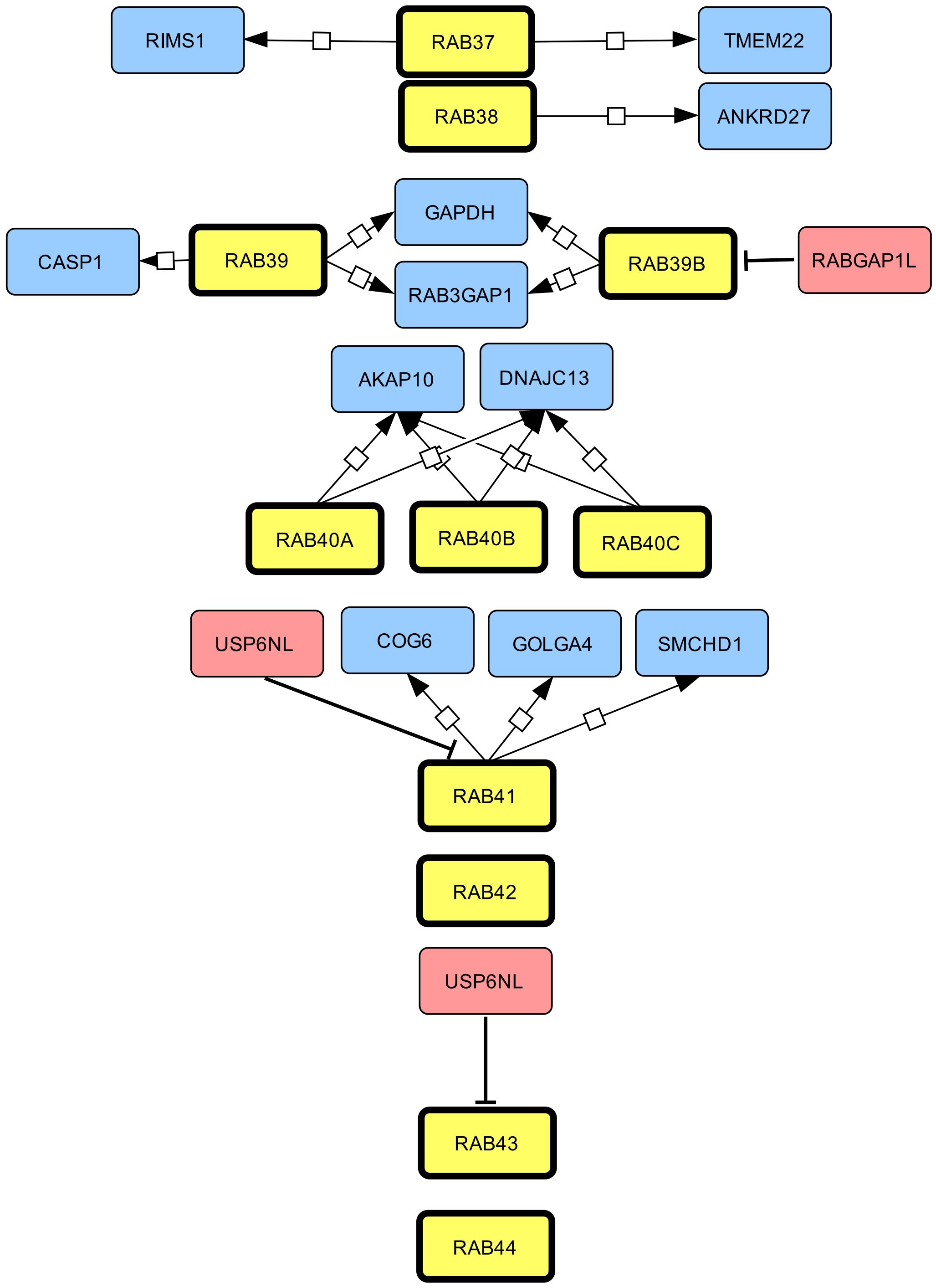
RIMS1
RAB37
TMEM22
RAB38
ANKRD27
GAPDH
CASP1
RAB39
RAB3GAP1
RAB39B
RABGAP1L
AKAP10
DNAJC13
RAB40A
RAB40B
RAB40C
USP6NL
COG6
GOLGA4
SMCHD1
RAB41
RAB42
USP6NL
RAB43
RAB44
