# Supplementary material for: Deregulation of Rab and Rab Effector Genes in Bladder Cancer
Source: PLoS One. 2012 Jun 19;7(6):e39469. doi: 10.1371/journal.pone.0039469 (PMC3378553; doi:10.1371/journal.pone.0039469)
Supplement: Table S2 — Genes deregulated during bladder cancer pathogenesis: second set of data. Second set of data. Affymetrix HG U95A/U95Av2 DNA microarrays. Genes down- or up-regulated in the tumor samples. Left: FGFR3-non-mutated tumor groups. Right: FGFR3-mutated tumor groups. The results shown to pass the thresholds: FC>1.5 (or <0.667) and qValue <5% with the first set of data are in red (or green) or highlighted in red (or green). The highlighted values in red or green pass the thresholds FC>1.5 or FC <0.667 or qValue <5% with the second set of data also. The results highlighted in yellow pass the thresholds: FC>1.5 (or <0.667) and qValue <5% only with the second set of data. (PDF) [file pone.0039469.s003.pdf]

Table S2. Genes deregulated during bladder cancer pathogenesis : Second set of data.

Non mutated *FGFR3* pathway: genes down regulated in tumoral samples

	TaG3/Normal		T1/Normal		T2T3T4/Normal	
	FC	qVal.(%)	FC	qVal.(%)	FC	qVal.(%)
**CASP1**	0.653	19.88	0.557	0	0.687	8.63
**CD2AP**	0.645	38.85	0.763	37.94	0.689	10.9
**EEA1**	0.917	69.57	0.835	31.99	0.761	2.33
**ICA1**	0.822	18.84	0.731	15.62	0.666	0.62
**PIGR**	0.691	46.53	0.593	7.30	0.554	0.29
**RAB11A**	0.614	18.84	0.818	27.39	0.651	3.05
**RAB14**	0.577	3.27	0.774	27.39	0.787	5.26
**RAB4A**	0.848	38.85	1.079	64.91	0.879	22.58
**TBC1D30**	0.850	50.58	0.714	4.01	0.699	1.33
**TBC1D4**	0.769	33.79	0.805	23.70	0.712	2.33

Non mutated *FGFR3* pathway: genes up regulated in tumoral samples

	TaG3/Normal		T1/Normal		T2T3T4/Normal	
	FC	qVal.(%)	FC	qVal.(%)	FC	qVal.(%)
**CAV1**	2.121	4.52	5.449	4.47	7.654	0
**ITGA5**	1.377	18.84	1.645	7.30	2.311	0.62
**MICAL2**	1.013	62.56	1.325	51.61	1.563	2.33
**RAB31**	0.860	65.81	0.979	58.9	1.488	20.03
**RABAC1**	1.156	41.97	1.432	6.55	1.603	0.81
**STXBP1**	1.237	41.97	1.134	63.56	1.415	25.39
**ZWINT**	1.977	33.79	1.726	59.42	2.251	3.05

Mutated *FGFR3* pathway: genes down regulated in tumoral samples

	TaG1G2/Normal		T1/Normal		T2T3T4/Normal	
	FC	qVal.(%)	FC	qVal.(%)	FC	qVal.(%)
**EEA1**	0.828	10.90	0.828	4.99	0.793	14.31
**ICA1**	0.892	10.90	0.877	8.88	0.775	0.89
**PIGR**	0.572	0	0.416	0	0.561	2.33
**RAB11FIP2**	0.848	15.08	1.017	29.50	0.691	0.89
**RAB14**	0.834	20.52	0.961	19.23	0.554	11.25
**RAB27A**	1.032	52.57	0.908	6.84	0.949	46.69
**RAB27B**	0.752	5.48	0.618	0.29	0.610	1.23
**RAB9A**	0.898	26.83	0.985	19.23	0.825	23.75
**RABGAP1L**	0.862	12.77	1.038	29.50	0.841	33.69
**TBC1D30**	0.832	1.19	0.700	0.16	0.774	5.40
**UNC13B**	0.629	0.36	0.666	0.12	0.665	0

Mutated *FGFR3* pathway: genes up regulated in tumoral samples

	TaG1G2/Normal		T1/Normal		T2T3T4/Normal	
	FC	qVal.(%)	FC	qVal.(%)	FC	qVal.(%)
**CAV1**	2.346	0.48	2.946	0	3.519	1.43
**ITGA5**	1.158	20.52	1.068	26.53	1.496	9.49
**SDC1**	1.491	0	1.491	0.42	1.648	11.25

Second set of data. Affymetrix HG U95A/U95Av2 DNA microarrays. Genes down- or up-regulated in the tumoral samples. Left: Non mutated *FGFR3* tumoral groups. Right: Mutated *FGFR3* tumoral groups. The results shown to pass the thresholds: FC > 1.5 (or < 0.667) and qValue < 5% with the first set of data are in red (or green) or highlighted in red (or green). The highlighted values in red or green pass the thresholds FC > 1.5 or FC < 0.667 or qValue < 5% with the second set of data also. The results highlighted in yellow pass the thresholds: FC > 1.5 (or < 0.667) and qValue < 5% only with the second set of data.
