# Supplementary material for: Deregulation of Rab and Rab Effector Genes in Bladder Cancer
Source: PLoS One. 2012 Jun 19;7(6):e39469. doi: 10.1371/journal.pone.0039469 (PMC3378553; doi:10.1371/journal.pone.0039469)
Supplement: Table S3 — Genes deregulated only in FGFR- non-mutated (A) and mutated tumors (B). Genes up- (or down)-regulated that pass the thresholds: FC>1.5 (or <0.667) and qValue <5% only for the FGFR3-non-mutated (A) or FGFR3-mutated (B) tumor pathway. Left column: comparison with the normal urothelium samples. Right column: comparison with the tumor samples of same stage with the opposite FGFR3 mutation status. The results that pass the above thresholds are highlighted (red for up-regulation and green for down-regulation). (PDF) [file pone.0039469.s004.pdf]

Table S3. Genes deregulated only in non mutated (A) and mutated FGFR3 tumors (B).

A)

	TaG3(*FGFR3* wt)/Normal		TaG3(*FGFR3* wt)/TaG1G2(*FGFR3* mutated)	
	FC	qVal.(%)	FC	qVal.(%)
GCC2	0.621	3.12	0.699	16.31

	T1(*FGFR3* wt)/Normal		T1(*FGFR3* wt)/T1(*FGFR3* mutated)	
	FC	qVal.(%)	FC	qVal.(%)
CASP1	0.256	2.06	0.832	63.93
RAB38	0.277	1.15	0.515	8.36
TBC1D4	0.437	3.35	0.740	29.45
TRAPPC1	0.527	4.46	0.751	22.66
KIF20A	4.024	4.46	1.368	63.93
LEPRE1	1.629	0.77	1.098	65.97
ZWINT	3.494	3.35	1.245	74.17

	T2T3T4(*FGFR3* wt)/Normal		T2T3T4(*FGFR3* wt)/T2T3T4(*FGFR3* mutated)	
	FC	qVal.(%)	FC	qVal.(%)
CD2AP	0.632	3.68	0.797	21.84
GCC2	0.610	3.04	0.782	21.84
RAB11A	0.498	1.41	0.717	13.93
RAB4A	0.541	3.68	0.724	19.79
SYTL1	0.339	2.68	0.418	1.72
LEPRE1	2.183	0	1.528	1.24
MICAL2	2.139	2.16	1.804	1.24
RAB23	2.674	2.68	1.920	4.08
RAB31	2.801	2.68	1.784	10.00
STXBP1	3.444	2.16	2.314	2.53
TMEM22	2.399	3.04	1.257	62.5
ZWINT	3.379	3.68	1.221	72.28

B)

	TaG1G2(*FGFR3* mutated)/Normal		TaG1G2(*FGFR3* mutated)/TaG3(*FGFR3* wt)	
	FC	qVal.(%)	FC	qVal.(%)
ANKRD27	0.648	1.64	0.691	19.14
RAB11FIP1	0.539	0.60	0.803	66.72
RAB11FIP2	0.572	3.25	0.956	71.66
RAB20	0.513	0.60	1.289	45.17
RAB27A	0.542	0.30	1.063	62.56
RAB27B	0.486	0	0.807	33.59
RAB8B	0.583	0.77	0.979	71.66
RAB9A	0.617	0.36	0.830	57.47
RABGAP1L	0.619	4.35	0.775	67.21
UNC13B	0.572	0.77	1.461	33.59

	T1(*FGFR3* mutated)/Normal		T1(*FGFR3* mutated)/T1(*FGFR3* wt)	
	FC	qVal.(%)	FC	qVal.(%)
GNAL	0.661	2.35	0.870	50.26
RAB20	0.484	4.55	0.751	28.39
RAB9A	0.582	2.88	0.881	71.93

	T2T3T4(*FGFR3* mutated)/Normal		T2T3T4(*FGFR3* mutated)/T2T3T4(*FGFR3* wt)	
	FC	qVal.(%)	FC	qVal.(%)
RAB11FIP2	0.521	1.63	0.795	55.97
RAB27B	0.469	1.16	0.927	76.07
RAB9A	0.591	4.45	0.804	45.50
UNC13B	0.502	1.63	0.857	71.38

Genes up- (or down)-regulated that pass the thresholds: FC > 1.5 (or < 0.667) and qValue < 5% only for the non mutated *FGFR3* (A) or mutated *FGFR3* (B) pathway. Left column: comparison with the normal urothelium samples. Right column: comparison with the tumoral samples of same stage with the opposite *FGFR3* mutation status. The results that pass the above thresholds are highlighted (red for up-regulation and green for down-regulation).
