# Supplementary material for: Deregulation of Rab and Rab Effector Genes in Bladder Cancer
Source: PLoS One. 2012 Jun 19;7(6):e39469. doi: 10.1371/journal.pone.0039469 (PMC3378553; doi:10.1371/journal.pone.0039469)
Supplement: Table S5 — Pearson correlation (r) (and pValue) between the expression of MKI67 and the expression of genes listed in left column. Pearson correlation (r) (and pValue) between the expression of MKI67 and the expression of genes listed in left column in 28 Ta G1G2 (FGFR3-mutated) tumor samples and 63 T2-4 (FGFR3-non-mutated) tumor samples. Are highlighted (in green or red) when |r| >0.479 for the Ta G1G2 (FGFR3-mutated) group (28 samples) and |r| >0.323 for the T2–4 (FGFR3-non-mutated) group (63 samples). (PDF) [file pone.0039469.s006.pdf]

Table S5. Pearson correlation (r) (and pValue) between the expression of *MKI67* and the expression of genes listed in left column.

Genes names	TaG1G2 (*FGFR3* mutated) (28 samples)		T2T3T4 (*FGFR3* wt) (63 samples)	
	Pearson correlation (r)	pValue	Pearson correlation (r)	pValue
MKI67	1		1	
ANKRD27	-0.197	0.314998	0.138	0.280776
CASP1	-0.193	0.325120	0.015	0.907113
CAV1	-0.276	0.155126	-0.151	0.237478
CD2AP	-0.19	0.332841	0.02	0.876363
EEA1	0.115	0.560083	-0.14	0.273795
GCC2	-0.109	0.580861	-0.285	0.023574
GNAL	-0.272	0.161444	-0.31	0.013416
ICA1	-0.549	0.002483	-0.197	0.121734
ITGA5	0.053	0.788814	-0.389	0.001628
KIF20A	0.86	0.000000	0.861	0.000000
LEPRE1	-0.381	0.045466	0.13	0.309869
MICAL1	-0.519	0.004656	-0.19	0.135830
MICAL2	0.206	0.292944	0.054	0.674240
MLPH	-0.069	0.727177	-0.425	0.000517
MYO5B	0.135	0.493388	0.096	0.454189
MYO5C	-0.623	0.000399	-0.477	0.000077
PIGR	0.004	0.983883	-0.434	0.000380
RAB11A	0.126	0.522897	-0.006	0.962776
RAB11FIP1	-0.083	0.674561	-0.306	0.014729
RAB11FIP2	-0.336	0.080444	-0.099	0.440143
RAB14	-0.256	0.188543	0.291	0.020678
RAB20	0.115	0.560083	-0.142	0.266931
RAB23	0.061	0.757814	-0.436	0.000354
RAB27A	0.361	0.059113	-0.183	0.151120
RAB27B	0.278	0.152034	0.153	0.231250
RAB31	-0.009	0.963746	0.027	0.833627
RAB38	-0.539	0.003081	0.204	0.108782
RAB4A	0.102	0.605522	-0.303	0.015784
RAB8B	0.058	0.769399	0.126	0.325118
RAB9A	-0.352	0.066212	0.014	0.913281
RABAC1	-0.055	0.781032	0.054	0.674240
RABGAP1L	-0.08	0.685720	-0.127	0.321261
RPH3AL	-0.304	0.115774	-0.111	0.386450
SDC1	0.055	0.781032	0.003	0.981383
STXBP1	-0.042	0.831948	-0.085	0.507743
SYTL1	-0.099	0.616224	-0.141	0.270349
SYTL2	-0.4	0.034939	-0.524	0.000010
TBC1D30	0.001	0.995971	0.053	0.679943
TBC1D4	-0.424	0.024539	-0.151	0.237478
TMEM22	0.135	0.493388	0.301	0.016523
TRAPPC1	-0.014	0.943631	0.083	0.517815
UNC13B	-0.336	0.080444	-0.225	0.076240
ZWINT	0.818	0.000000	0.841	0.000000

Pearson correlation (r) (and pValue) between the expression of *MKI67* and the expression of genes listed in left column in 28 Ta G1G2 (*FGFR3* mutated) tumoral samples and 63 T2T3T4 (*FGFR3* wt) tumoral samples. Are highlighted (in green or red) when $|r| > 0.479$ for the Ta G1G2 (*FGFR3* mutated) group (28 samples) and $|r| > 0.323$ for the T2T3T4 (*FGFR3* wt) group (63 samples).
